# Perfluoroalkyl substances (PFASs) decrease the expression of recombination-activating genes (*RAG1* and *RAG2*) in human B lymphoma Namalwa cells

**DOI:** 10.1007/s00204-022-03405-z

**Published:** 2022-11-03

**Authors:** Aafke W. F. Janssen, Jochem Louisse, Deborah Rijkers, Nicole E. T. Pinckaers, Sjoerdtje A. Hoekstra, Ron L. A. P. Hoogenboom, Ad A. C. M. Peijnenburg, Karsten Beekmann

**Affiliations:** Wageningen Food Safety Research (WFSR), Wageningen University and Research, Akkermaalsbos 2, 6708 WB Wageningen, The Netherlands

**Keywords:** PFASs, Immunotoxicity, Namalwa cells, RNA sequencing

## Abstract

Per- and polyfluoroalkyl substances (PFASs) are omnipresent and have been shown to induce a wide range of adverse effects, including hepatotoxicity, developmental toxicity and immunotoxicity. So far, little information is available about the mechanisms underlying the toxicity of PFASs, including those related to their immunotoxicity. Reported immunotoxic effects of PFASs include decreased antibody responses in experimental animals and humans, indicating that PFASs may, among others, affect B cell function. In the present study, we first assessed the effects of PFOA on the transcriptome of the human Namalwa B cell line using RNA seq analysis. Gene expression changes, analyzed using Ingenuity Pathway Analysis, pointed to various cellular processes affected by PFOA, including ‘B cell development’ and ‘Primary immunodeficiency signaling’. Interestingly, PFOA decreased the expression of *RAG1* and *RAG2*, genes involved in immunoglobulin and T cell receptor V(D)J recombination. As a next step, time- and concentration-dependent changes in the expression of *RAG1* and *RAG2* upon exposure to PFOA, PFNA, PFHxS and PFOS were studied through RT-qPCR analysis. Analysis with the concentration–response modeling software PROAST resulted in the following potency ranking: PFNA > PFOA > PFOS > PFHxS. Altogether, the present in vitro study provides insights into the effects of selected PFASs on B cells, identifying *RAG1* and *RAG2* expression as possible relevant targets that may play a role in the immunotoxicity of PFASs.

**Supplementary Information:**

The online version contains supplementary material available at 10.1007/s00204-022-03405-z.

## Introduction

Per- and polyfluoroalkyl substances (PFASs) are anthropogenic chemicals that are omnipresent in the environment and can end up in food or drinking water (Wang et al. 2017). PFASs contain a fluorinated carbon chain of different length, with diverse functional groups attached at the end of the chain. Because of this chemical structure, PFASs are extremely persistent and have unique physical and chemical properties. They are widely used in various industrial and consumer applications, including firefighting foams, electronics, textiles, food contact materials, and cosmetics. Given the concerns of adverse effects to human health and the environment, the production and use of the most studied PFASs, perfluorooctanoic acid (PFOA) and perfluorooctane sulfonic acid (PFOS), have been restricted (EFSA CONTAM Panel 2018, 2020; ATSDR 2021).

**Fig. 1:**
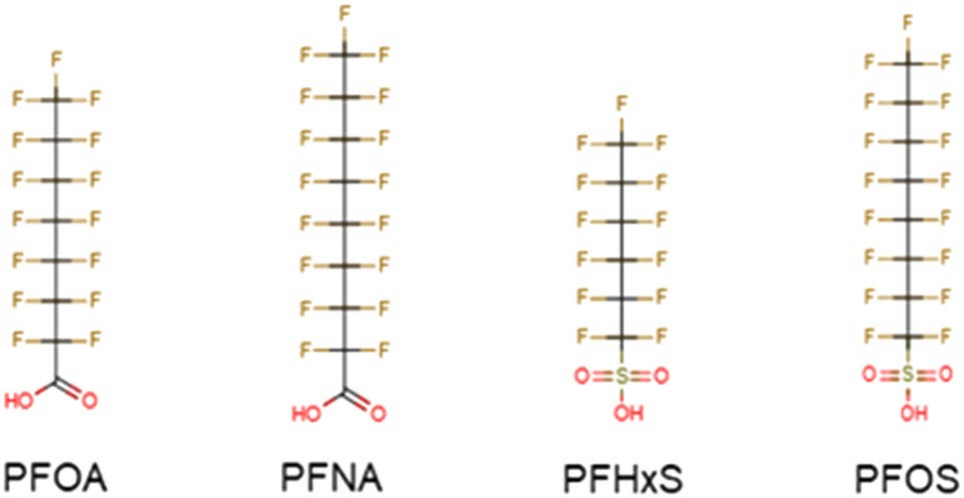
Chemical structures of the PFASs tested in the present study. *PFOA* perfluorooctanoic acid, *PFNA* perfluorononanoic acid, *PFHxS* perfluorohexane sulfonic acid, *PFOS* perfluorooctane sulfonic acid

PFASs have been shown to induce a wide range of adverse effects, including hepatotoxicity, developmental toxicity, a decrease in thyroid hormone levels, and immunotoxicity (EFSA CONTAM Panel 2020). In its most recent opinion on PFASs, the EFSA CONTAM panel established a health based guidance value (tolerable weekly intake (TWI) of 4.4 ng/kg bw) for four PFASs (PFOA, PFOS, PFNA, PFHxS; Fig. 1) based on human data showing a negative association between the levels in blood of PFOA, but also the sum of 4 PFASs, and antibody titres against diphtheria (Abraham et al. 2020; EFSA CONTAM Panel 2020). Data on serum levels of these four PFASs in 1-year-old breastfed infants were used to obtain an internal concentration as point of departure (amounting to 17.5 ng/mL), which was extrapolated to an external dose level applying physiologically based kinetic (PBK) modeling-facilitated reverse dosimetry (EFSA CONTAM Panel 2020). Since the main exposure of infants is in utero and especially via human milk, a safe body burden in mothers was estimated, and the long-term exposure resulting in such body burden as the basis of the new TWI, being much lower than the previous one established for effects on liver and thyroid in animals. No studies were identified to derive relative potency factors (RPFs) for effects of PFASs on the immune system.

Immunotoxicity of PFASs has also been observed in animal studies. Various studies reported a decrease of IgM or anti-sheep red blood cell plaque forming cells mediated by a decreased T cell-dependent antibody response (TDAR) (DeWitt et al. 2008, 2009, 2016; Peden-Adams et al. 2008; Dong et al. 2009, 2011; Zheng et al. 2009, 2011), or by a T cell-independent antibody response (TIAR) (Peden-Adams et al. 2008; Vetvicka and Vetvickova 2013; DeWitt et al. 2016), whereas others did not find such effects (Lefebvre et al. 2008; Qazi et al. 2010). Although various cellular processes affected by PFASs have been suggested to play a possible role in the mode-of-action underlying their immunotoxic effects (Liang et al. 2022), a clear understanding of the PFAS-induced decrease in antibody response is lacking. PFASs have been reported to activate PPARα (e.g., Behr et al. 2020; Evans et al. 2022) and, therefore, the role of PPARα in PFAS-induced immunotoxicity has been studied. Animal studies with wild-type and PPARα knockout mice indicate that the effect of PFOA on TDAR is not dependent on a functional PPARα (DeWitt et al. 2016). Since animal studies have shown that PFOA and PFOS reduced the antibody response in models for TIAR and TDAR, a direct effect of these PFASs on B cells may be expected.

Several in vitro B cell systems have been described that can be used as relevant models to screen substances for their modulatory effects on B cell activation and B cell development and for mode-of-action studies. Interesting in this regard is the study by Van Belle et al. (2016) who assessed the potential of different human B cell lines for investigating the effects of B lymphocyte immunosuppressants. The authors concluded that the Namalwa cell line can be considered as a relevant B cell model for the discovery of new targets and inhibitors of the B cell activation processes (Van Belle et al. 2016). Therefore, we considered the Namalwa cell line as a relevant human cell model to provide insights whether human B cells may be a relevant target for PFASs and may play a role in the observed decrease in antibody response in humans and animals. The present study aimed to assess the effects of PFOA, PFOS, PFNA, and PFHxS on human B cells, by analyzing gene expression changes in Namalwa B cells using RNA seq and RT-qPCR. In addition the dose–response curves were used to obtain in vitro RPFs for these four PFASs.

## Materials and methods

### Chemicals

The following PFASs were tested in the present study: perfluorooctanoic acid (PFOA), perfluorononanoic acid (PFNA), perfluorohexane sulfonate (PFHxS), and perfluorooctane sulfonate (PFOS). In addition, the effects of the FOXO1 inhibitor AS1842856 were studied. All stocks were prepared in 100% dimethyl sulfoxide (DMSO HybriMax, Sigma-Aldrich). More information about suppliers, purity, catalog numbers, CAS numbers and maximum concentrations tested in the present study is presented in Supplementary Table 1.

### Namalwa cell culture

The human Burkitt’s lymphoma cell line Namalwa was obtained from Sigma-Aldrich (Zwijndrecht, The Netherlands) and maintained in RPMI1640 (Gibco, Thermo Fisher Scientific, Waltham, MA) supplemented with 10% heat-inactivated fetal bovine serum (Gibco), 1% Sodium Pyruvate (Sigma–Aldrich), 1% NEAA (Gibco) and 1% penicillin–streptomycin (Sigma–Aldrich). Once the Namalwa cells were growing at a stable growth rate (approximately after 10 days), the amount of heat-inactivated fetal bovine serum was reduced to 2% to limit binding of PFASs to proteins to allow significant cell exposure. Namalwa cells were subcultured twice a week, each time diluted to 0.5 × 10^6^ viable cells/mL.

### Cell viability studies

The effects of the 4 PFASs and AS1842856 on Namalwa cell viability were determined using the WST-1 assay. This assay determines the conversion of the tetrazolium salt WST-1 (4-[3-(4-iodophenyl)-2-(4-nitrophenyl)-2H-5-tetrazolio]-1,3-benzene disulfonate) to formazan by metabolically active cells. Namalwa cells were cultured in 96-well plates (1 × 10^6 cells/ml) and exposed to increasing concentrations up to 100 µM for PFOA, PFOS, PFNA and PFHxS for 48 h or to increasing concentration up to 0.1 µM AS1842856 for 48 h. After exposure, WST-1 solution (Sigma-Aldrich) was added to the cell culture medium (1:10 dilution). After 1 h incubation in an incubator (humidified atmosphere with 5% CO_2_ at 37 °C), the plate was shaken at 1000 rpm for 1 min, and absorbance at 450 nm was measured (background absorbance at 630 nm was subtracted) using a Synergy HT Microplate Reader (BioTek, Winooski, VT). Cell viability upon PFAS and AS1842856 treatments was expressed as percentage of the cell viability of the solvent control. DMSO concentration was 0.1% in solvent control and in all treatment conditions.

### Namalwa exposure for gene expression analysis

For gene expression studies, Namalwa cells were seeded in 24-well plates (Corning, NY; 0.5–1 × 10^6 cells per well in 500 µL). Test chemicals were first diluted from a 1000-fold concentrated stock to a twofold concentrated stock solution in assay medium and subsequently twofold diluted upon the addition to the Namalwa cell suspension, providing a final DMSO concentration of 0.1%. In each experiment a solvent control (0.1% DMSO) was included. For the RNA seq study, Namalwa cells were exposed to 100 µM PFOA for 48 h. For RT-qPCR studies, Namalwa cells were exposed to either the highest non-cytotoxic concentration of 100 µM for PFOA, PFOS and PFHxS, and 33 µM for PFNA for 6, 24 and 48 h or to a concentration range up to 100 µM for PFOA, PFOS and PFHxS, and up to 33 µM for PFNA for 48 h. In a follow-up study, Namalwa cells were exposed to 0.0001, 0.001 and 0.01 µM AS1842856 for 6, 24 and 48 h. After exposure, effects of the PFASs and AS1842856 on expression of selected genes were assessed.

### RNA library preparations and RNA seq

To obtain insight into the PFOA-induced gene expression changes, Namalwa cells were exposed to 100 µM PFOA or solvent control for in total 48 h. After exposure, medium including cells were collected and centrifuged at 200 × g for 7 min to obtain a cell pellet. The Namalwa cell pellets were subsequently lysed in cell lysis buffer (RLT) and total RNA was isolated and purified using the RNeasy Mini Kit (Qiagen). Total RNA was quantified using Qubit (Life Technologies) and RNA integrity was analyzed using Agilent 2100 Bioanalyzer total RNA Pico chip (Agilent Technologies). Subsequent RNA Library preparations and RNA sequencing was performed at Genomics Facility of Wageningen University and Research, Business Unit Bioscience. Approximately, 1 µg total RNA was used for RNA library preparation using TruSeq Stranded mRNA Sample Prep kit (Illumina). In short, after polyA based mRNA selection, RNA were further processed including subsequent fragmentation, first and second strand cDNA synthesis, adapter ligation and final library amplification resulting in RNA seq libraries including unique dual indexes, all following manufacturer’s protocol. Final libraries were eluted in 30 µl elution buffer followed by library quality assessment using a Fragment Analyzer (Agilent Technologies) and quantified by Qubit fluorescence measurements (Invitrogen, Life Technologies).

Prepared libraries were pooled in an equimolar manner and combined with other indexed libraries for sequencing on an Illumina NovaSeq 6000 system. Final sequencing was done using an S2 and S4 type flow cell, both with XP loading workflow and settings specific for 2 × 150 nt paired end reads plus dual indexes reads. All steps for sequencing were carried out according to manufacturer’s protocol. Demultiplexing of reads per sample by corresponding indexes was done using bcl2fastq v2.20.0.422 (Illumina Inc, San Diego, CA, USA).

### Processing of RNA sequencing reads

The RNA seq reads were used to quantify transcript abundances. To this end, the tool Cutadapt (version 1.16) (Martin 2011) was used to trim adapters from the reads and HISAT2 (version 2.2.1) (Kim et al. 2019) was used to map the reads to the GRCm38.13 human genome assembly-based transcriptome sequences as annotated by the GENCODE consortium (release M40). HISAT2 output was converted and sorted by chromosomal position using Samtools (version 1.9) (Danecek et al. 2021). RSeQC (v3.0.1) (Wang et al. 2012) and PRINSEQ (v0.20.4) (Schmieder and Edwards 2011) were used for quality control. HTSeq (version 0.11.2) (Anders et al. 2015) was used to count reads in transcripts using gene-level quantification. Differential gene expression was determined using the package limma (version 3.50.3) (Ritchie et al. 2015) utilizing the obtained scaled gene-level counts. Briefly, before statistical analyses, nonspecific filtering of the count table was performed to increase detection power, based on the requirement that a gene should have an expression level greater than around 10 counts in at least three samples. Differences in library size were adjusted by the trimmed mean of M-values normalization method, implemented in the package edgeR (version 3.36.0) (Robinson et al. 2009; McCarthy et al. 2012; Chen et al. 2016). Counts were transformed to log2 (cpm) values and associated precision weights, and entered into the limma analysis pipeline. Differentially expressed genes were identified using generalized linear models that incorporate empirical Bayes methods.

### Ingenuity pathway analysis

Gene lists containing gene identifiers (Ensembl Gene ID), and corresponding log_2_ fold changes and p-values were uploaded to Ingenuity Pathway Analysis (IPA) software (Qiagen, Redwood City, CA, USA). Input criteria were a log_2_ fold change of above 0.5 and a *p* value below 0.01. To interpret biological meaning of differentially expressed genes, gene sets were analyzed using the Canonical Pathways module. Statistically overrepresented pathways were identified by Fisher’s exact test (*p* < 0.01).

### RT-qPCR

After exposure of Namalwa cells to PFASs, medium including cells were collected and centrifuged at 200 × g for 7 min to obtain a cell pellet. The Namalwa cell pellets were lysed in cell lysis buffer (RLT) and total RNA was extracted using the RNeasy Mini Kit (Qiagen, Venlo, The Netherlands). Subsequently, 500 ng RNA was used to synthesize cDNA using the iScript cDNA synthesis kit (Bio-Rad Laboratories, Veenendaal, The Netherlands). Changes in gene expression were determined by RT-qPCR on a CFX384 real-time PCR detection system (Bio-Rad Laboratories) using SensiMix (Bioline; GC Biotech, Alphen aan den Rijn, The Netherlands). The PCR conditions consisted of an initial denaturation of 95 °C for 10 min, followed by 40 cycles of denaturation at 95 °C for 10 s and annealing extension at 60 °C for 15 s. Primer sequences were taken from the Harvard PrimerBank and ordered from Eurogentec (Liège, Belgium). Sequences of the used primers are listed in Table 1. Relative gene expression was quantified with the standard curve method, using a standard curve generated from a serial dilution of pooled sample cDNA, and subsequently normalized to glyceraldehyde 3-phosphate dehydrogenase (GAPDH) gene expression. Gene expression upon PFAS treatments was expressed as fold change compared to the gene expression measured for the solvent control. The concentration–response data were subjected to BMD analysis using PROAST software as described as follows.

**Table 1:** Primer sequences used for RT-qPCR

	Primer sequence	
Name	Forward	Reverse
*GAPDH*	CTCTGCTCCTCCTGTTCGAC	TTAAAAGCAGCCCTGGTGAC
*RAG1*	TGCACAGGAAGTTTAGCAGTG	ACGGGCAGTGTTGCAGATG
*RAG2*	AGACTTGGTAGGAGATGTTCCTG	TGTATGAGCGTCCTCCAAAGAG

### Benchmark dose (BMD) analysis of RT-qPCR data using PROAST

RT-qPCR data were used for concentration–response modeling with BMD analysis software PROAST version 70.5 (National Institute for Public Health and the Environment 2018) in R (version 4.2.0). PROAST is particularly applied for modeling of in vivo (dose–response) data, providing information on the BMD. In the present work, PROAST software was used for the analysis of in vitro (concentration–response) data, thereby providing information on the benchmark concentration (BMC). Data of all PFASs were analyzed simultaneously to ensure the parallel curves required to derive RPFs. Tab-delimited text files containing data on concentration, experiment number, mean effect, standard deviation, and sample size (number of replicates) were made and analyzed as continuous (summary) data. For PROAST analysis, average gene expression values of triplicates with standard deviation of two independent studies were used (Supplementary file 2). Independent studies were assigned as covariates. Then, the exponential model:

$$y=a*{c}^{1-exp\left(-{\left(x/b\right)}^{d}\right)}$$

with y denoting the response and x the concentration was applied. The parameters a, b, c, and d describe the response at dose 0 (background value), the potency of the PFAS, maximum fold change in response compared with background response (upper or lower plateau), and steepness of the curve (on a log-dose scale), respectively. BMC values were determined for a benchmark response of 10% (BMR_10_) for the RT-qPCR data. The model with the lowest Akaike information criterion (AIC) was chosen to determine RPF values including 90% confidence intervals. PFOA was used as the index chemical. Of note, PROAST definitions are CES (critical effect size) and CED (critical effect dose), which are the same as BMR and BMC, respectively.

### Statistical analysis

Data are presented as mean ± SD. A one-way ANOVA followed by Dunnett’s post hoc multiple comparison test was used for comparisons between Namalwa cells exposed to various concentrations of AS1842856 and solvent control. *p* < 0.05 was considered as statistically significant. Prism software (version 9.2.0; Graphpad, San Diego, CA) was used for statistical analysis.

## Results

### Cell viability studies

**Fig. 2:**
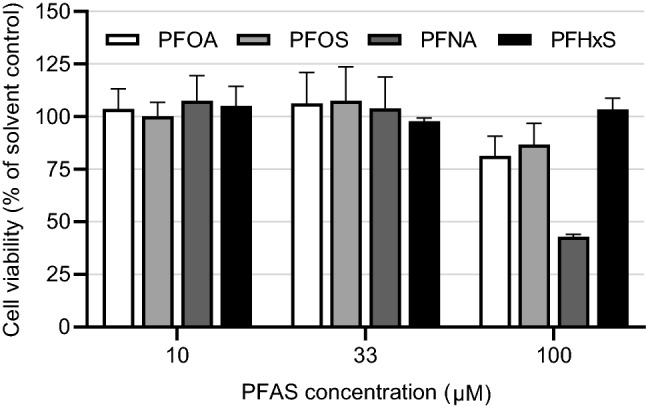
Effects of 48 h exposure to PFOA, PFOS, PFNA and PFHxS on viability of Namalwa cells. Viability was determined using the WST-1 assay and expressed as percentage of the solvent control (0.1% DMSO). Data presented as mean ± SD from 6 wells (data from two independent runs combined)

Namalwa cells were exposed to increasing concentrations of PFASs (up to 100 µM) for 48 h to determine cell viability using the WST-1 assay. Of the 4 tested PFASs, PFNA was the most cytotoxic giving more than 50% reduction in cell viability 100 µM (Fig. 2). Based on these data, the highest concentrations were selected for gene expression studies, amounting to 100 µM PFOA, PFOS and PFHxS and 33 µM PFHxS, being concentrations causing not more than 20% reduction in cell viability.

### Transcriptomics effects of PFOA

**Fig. 3:**
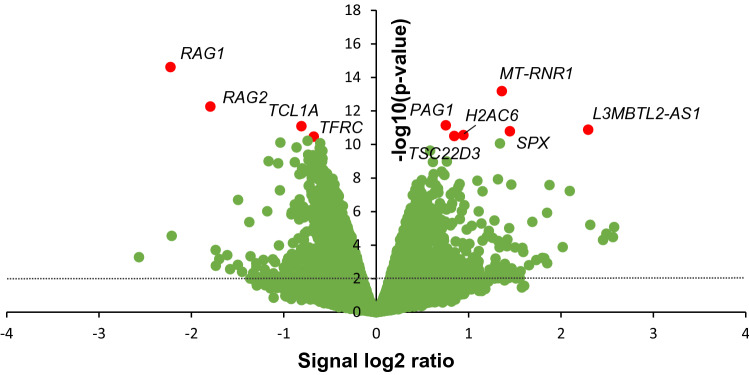
Effect of PFOA on gene expression in Namalwa cells. Volcano plot showing relative changes in gene expression (expressed as signal log_2_ ratio, x-axis) plotted against statistical significance (expressed as -log10 *p* value of empirical Bayes moderated *t-*statistic *p* value, y-axis). The 10 most significantly regulated genes are highlighted in red

To get more insight into the direct effects of PFOA on human B cells, Namalwa cells were exposed to 100 µM PFOA for a duration of 48 h, and subjected to genome-wide transcriptome analysis using RNA sequencing. Using a statistical cutoff of *p* < 0.01 (empirical Bayes moderated t-statistic) and a log_2_ fold change of > 0.5, PFOA altered the expression of 574 genes in Namalwa cells. Of these 574 genes, 279 genes were found to be upregulated and 295 genes were downregulated (Fig. 3, Supplementary Table 2). The top 10 most significantly regulated genes are highlighted in Fig. 3 and listed in Table 2. Of the ten most significantly regulated genes, six genes were upregulated and four genes were downregulated. The most significantly upregulated gene was *MT-RNR1*, a ribosomal RNA gene involved is osteoblast proliferation, carbohydrate utilization and various metabolic processes, followed by *PAG1* (a type III transmembrane adaptor protein which is thought to negatively regulate T cell activation), *L3MBTL2-AS1* (a long non-coding RNA)*, SPX* (spexin hormone involved in metabolism, energy homeostasis and reproduction), *H2AC6* (a core component of the nucleosome) and *TSC22D3* (a protein having a key role in anti-inflammatory and immunosuppressive effects of glucocorticoids). The four most significantly downregulated genes by PFOA were *RAG1*, *RAG2*, *TCL1A* and *TFRC*. *TFRC* is a cell surface receptor involved in the cellular uptake of iron and *TCL1A* is expressed in immature T and B lymphoid cells involved in promoting cell survival, growth and proliferation. Interestingly, PFOA downregulated the expression of both *RAG1* and *RAG2* showing a *p* value of 2.44 × 10^–15^ and 5.53 × 10^–13^, respectively. *RAG1* and *RAG2* encode lymphoid-specific proteins that are essential for V(D)J recombination leading to the generation of unique sets of immunoglobulins and T cell receptors.

**Table 2:** The ten most significantly regulated genes in Namalwa cells upon PFOA treatment

Gene symbol	Description	*p* value	Fold change	Ensembl gene ID
*RAG1*	Recombination activating gene 1	2.44 E-15	− 4.68	ENSG00000166349
*MT-RNR1*	Mitochondrially encoded 12S ribosomal RNA	6.64 E-14	2.57	ENSG00000211459
*RAG2*	Recombination activating gene 2	5.53 E-13	− 3.47	ENSG00000175097
*PAG1*	Phosphoprotein membrane anchor with glycosphingolipid microdomains 1	7.23 E-12	1.69	ENSG00000076641
*TCL1A*	T cell leukemia/lymphoma 1A	8.12 E-12	− 1.75	ENSG00000100721
*L3MBTL2-AS1*	L3MBTL2 antisense RNA 1	1.33 E-11	4.91	ENSG00000235513
*SPX*	Spexin hormone	1.64 E-11	2.73	ENSG00000134548
*H2AC6*	H2A clustered histone 6	2.79 E-11	1.93	ENSG00000180573
*TSC22D3*	TSC22 domain family member 3	3.15 E-11	1.80	ENSG00000157514
*TFRC*	Transferrin receptor	3.45 E-11	− 1.60	ENSG00000072274

### Ingenuity pathway analysis

To gain better insight into the biological function of the genes modified by PFOA in Namalwa cells, Ingenuity Pathway Analysis (*p* < 0.01) was performed for the differentially expressed genes with a log_2_ fold change of > 0.5. 31 canonical pathways were found to be modulated in Namalwa cells upon PFOA treatment with a threshold of *p* < 0.01 (Supplementary Fig. 1). Differentially expressed genes in the Namalwa cells treated with PFOA associated with each pathway are listed in Supplementary File 3. The most significantly changed pathway was ‘Kinetochore Metaphase Signaling’ (downregulated). Interestingly, PFOA affected two cellular processes related to immune function, including ‘B Cell Development’ and ‘Primary Immunodeficiency Signaling’. The genes in this data set associated with B Cell Development are *CD79B*, *HLA-DQA2*, *IL7R*, *RAG1* and *RAG2*. Primary immunodeficiency signaling was represented by downregulation of *AICDA*, *IGLL1*/*IGLL5*, *IL7R*, *RAG1* and *RAG2*. As mentioned above, *RAG1* and *RAG2* are the most highly downregulated genes by PFOA in Namalwa cells showing a *p* value of 2.44 × 10^–15^ and 5.53 × 10^–13^, respectively (Table 2). Taken together, particularly considering the affected immune pathways, these data indicate that downregulation of RAG expression is possibly playing a role in PFOA-induced effects on B cell function.

### Effects of PFOA, PFNA, PFHxS and PFOS on RAG1 and RAG2 expression

**Fig. 4:**
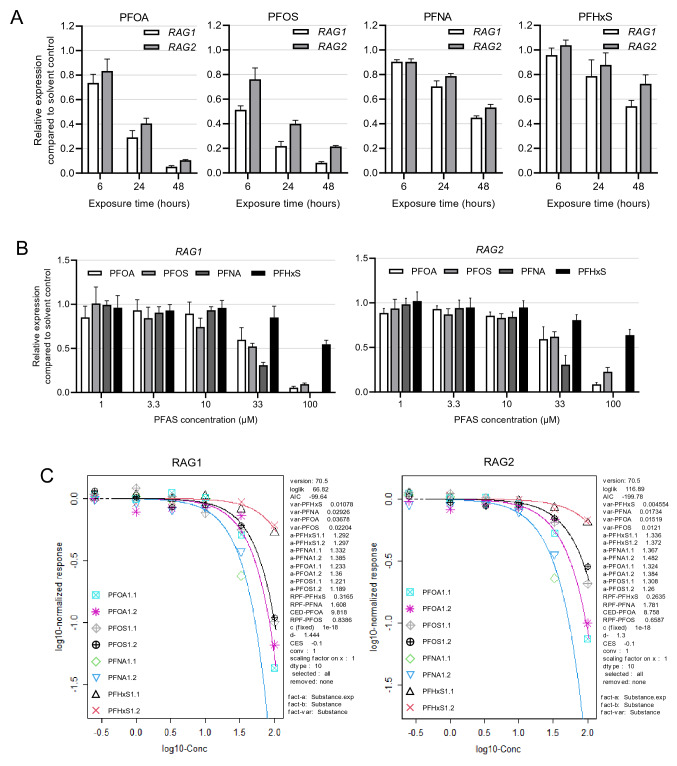
Time- and concentration-dependent effects of PFOA, PFOS, PFNA and PFHxS on *RAG1* and *RAG2* expression in Namalwa cells. **A** Relative expression of *RAG1* and *RAG2* after incubation of Namalwa cells with 100 µM PFOA, 100 µM PFOS, 33 µM PFNA or 100 µM PFHxS for 6, 24 or 48 h. Gene expression levels of the solvent controls were set at one. Data are mean values ± SD from triplicate wells. **B** *RAG1* and *RAG2* gene expression after treatment of Namalwa cells for 48 h with increasing concentrations of PFOA, PFOS, PFNA and PFHxS. Gene expression levels of the solvent controls were set at one. The data points represent the mean ± SD of 2 independent studies, each performed in triplicate (*n* = 6). **C** Results of BMD modeling using concentration-effect data regarding *RAG1* and *RAG2* gene expression, generated by PROAST for a CES of -0.1, which corresponds to a BMR of 10% (BMR_10_). The observed changes in *RAG1/2* expression in response to the PFASs appeared to be best described by the exponential model $y=a*{c}^{1-exp\left(-{\left(x/b\right)}^{d}\right)}$, containing the lowest Akaike information criterion (AIC). The used parameters were a, b, c, and d describing the response at dose 0 (background value), the potency of the PFAS, maximum fold change in response compared with background response (upper or lower plateau), and steepness of the curve (on a log-dose scale), respectively. CES: critical effect size (same as BMR), CED: critical effect dose (same as BMC), CEDL: lower bound of the CED (same as BMCL), CEDU: upper bound of the CED (same as BMCU). Data points represent the mean of triplicates, showing data from two independent studies for each PFAS

To assess whether *RAG1* and *RAG2* are also downregulated upon PFOS, PFNA and PFHxS exposure, first a time-course experiment was conducted in which Namalwa cells were exposed for 6, 24 and 48 h to 100 µM PFOA, 100 µM PFOS, 33 µM PFNA or 100 µM PFHxS followed by *RAG1* and *RAG2* gene expression analysis using RT-qPCR. All PFASs caused a time-dependent reduction in *RAG1* and *RAG2* expression (Fig. 4A). Given that the highest reduction in *RAG1* and *RAG2* expression was obtained after 48 h, this exposure duration was applied in follow-up experiments. To gain insight into relative potencies of the four PFASs, Namalwa cells were exposed to a concentration range up to 100 µM for PFOA, PFOS and PFHxS, and up to 33 µM for PFNA followed by *RAG1* and *RAG2* gene expression analysis. Figure 4B shows that all four PFASs caused a concentration-dependent reduction in *RAG1* and *RAG2* gene expression in Namalwa cells. Subsequently, expression data were used to perform concentration–response modeling using PROAST software (as described in Materials and methods section) to determine possible differences in potencies between the PFASs. To that end, benchmark concentrations (BMCs) corresponding to a 10% reduction in *RAG1* and *RAG2* expression compared to background level, were determined, applying parallel curve fitting. Results from the dose–response modeling for *RAG1* and *RAG2* are presented in Fig. 4C with corresponding BMC_10_ values in Table 3. RPFs determined by PROAST are also presented in Table 3. Although only slight differences in relative potencies were found, PFNA turned out to be the most potent of the four PFASs, being 1.6-fold and 1.8-fold more potent than PFOA in reducing *RAG1* and *RAG2* expression, respectively. PHFxS was the least potent, being 3.1-fold and 3.8-fold less potent than PFOA in reducing *RAG1* and *RAG2* expression, respectively. PFOS’s potency was slightly lower than that of PFOA, i.e., 0.8-fold lower for *RAG1* and 0.7-fold lower for *RAG2* (Table 3).

**Table 3:** Overview of BMC_10_ values (in µM) and related RPFs determined upon BMD modeling of data on reduction in RAG1 and RAG2 gene expression

	*RAG1*		*RAG2*	
PFAS	BMC_10_	RPF	BMC_10_	RPF
PFOA	9.8 (7.4–12.9)	1^a^	8.8 (6.6–11.0)	1^a^
PFOS	11.7 (8.9–15.2)	0.84 (0.8–0.9)	13.3 (10.6–16.2)	0.66 (0.6–0.7)
PFNA	6.1 (4.9–7.6)	1.61 (1.4–1.8)	4.9 (4.0–6.0)	1.78 (1.6–1.9)
PFHxS	31.0 (25.9–36.7)	0.32 (0.3–0.4)	33.2 (28.5–38.4)	0.26 (0.2–0.3)

^a^PFOA is used as an index chemical and potency was set at 1

90% confidence intervals are presented between brackets

### Effect of FOXO1 inhibitor AS1842856 on RAG1 and RAG2 expression in Namalwa cells

**Fig. 5:**
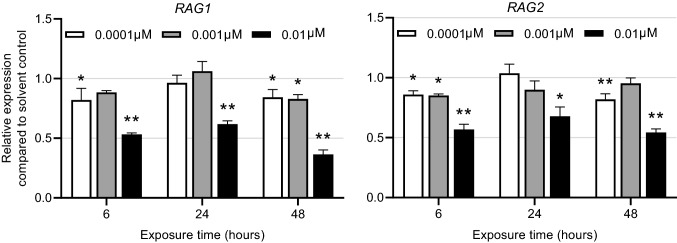
FOXO1 inhibitor AS1842856 downregulates *RAG1* and *RAG2* expression. Relative expression of *RAG1* and *RAG2* after incubation of Namalwa cells with 0.0001, 0.001 or 0.01 µM AS1842856 for 6, 24 and 48 h. *P < 0.05, **P < 0.001. Gene expression levels of the solvent controls were set at one. Data are mean ± SD from triplicate wells

*RAG1* and *RAG2* are target genes of the transcription factor FOXO1 and inactivation of FOXO1 has been described to reduce expression of *RAG1* and *RAG2* (Lazorchak et al. 2010; Lazorchak and Su 2011; Benhamou et al. 2018; Peña-Pérez et al. 2022). In order to study whether inhibition of FOXO1 also downregulates *RAG1* and *RAG2* expression in Namalwa cells, they were exposed to increasing concentrations (up to 0.1 µM) of the FOXO1 inhibitor AS1842856 for 6, 24 and 48 h. After exposure to 0.01 and 0.1 µM, Namalwa cells were subjected to the WST-1 cell viability assay, showing no effect of AS1842856 on cell viability at 0.01 µM and a reduction in cell viability at 0.1 µM (Supplementary Fig. 2). Effects on gene expression were studied at 0.0001, 0.001 and 0.01 µM AS1842856. Limited to no effects of AS1842856 were observed at 0.0001 and 0.001 µM, whereas 0.01 µM AS1842856 caused a reduction of *RAG1* and *RAG2* expression at all time points (Fig. 5). This indicates that *RAG1* and *RAG2* expression in Namalwa cells is regulated by FOXO1 and points to a possible PFAS-induced inhibition of FOXO1 resulting in decreased expression of *RAG1*/*RAG2*.

## Discussion

The present study aimed to assess the effects of PFOA, PFNA, PFHxS and PFOS on the human B cell line Namalwa using gene expression studies, to obtain more mechanistic insight into the PFAS-induced reduced antibody response in experimental animals and humans. RNA seq analyses of PFOA-exposed Namalwa cells showed various genes to be regulated and Ingenuity Pathway Analysis of these data showed various pathways to be affected, including pathways related to immune function (i.e., ‘B Cell Development’ and ‘Primary immunodeficiency signaling’). Of the genes showing the largest effects upon PFOA treatment, *RAG1* and *RAG2* are part of the modulated pathways related to immune function. Therefore, these genes were considered relevant to also assess the effects of the other PFASs (PFNA, PFHxS and PFOS) to obtain insight into possible potency differences. Concentration–response modeling of the *RAG1* and *RAG2* gene expression data using parallel curve fitting in PROAST, revealed that PFNA showed the highest potency regarding *RAG1/RAG2* downregulation, whereas PFOS and especially PFHxS were less potent than PFOA.

The present study shows that PFOA, PFOS, PFNA and PFHxS cause a time- and concentration-dependent downregulation of *RAG1* and *RAG2* gene expression in the human B cell lymphoma Namalwa cell line. RAG enzymes are lymphoid-specific proteins that have a crucial role in the maturation of T and B lymphocytes. RAG enzymes initiate V(D)J recombination at the variable region of the antigen receptor allowing for the generation of T and B lymphocytes with a diverse repertoire of T cell receptors and immunoglobulins, respectively. The RAG complex consists of a heterotetramer with two subunits of each RAG1 and RAG2 which introduces double strand DNA breaks at specific recombination signal sequences that flank each V (variable), D (diversity) and J (joining) gene segment (Bassing et al. 2002; Schatz and Ji 2011). RAG1 directly binds to the DNA, where it nicks the DNA and subsequently creates a double strand break. RAG2 has no direct contribution to DNA cleavage, but stabilizes and confers specificity of the RAG1 interaction with DNA, thereby enhancing RAG1’s cleaving activity by more than 100-fold (Mo et al. 1999; Gan et al. 2021). After DNA cleavage, DNA repair enzymes randomly assemble one of each V, D and J gene segments through non-homologous end joining, in order to generate an almost limitless set of T cell receptors and immunoglobulins (Bassing et al. 2002; Schatz and Ji 2011). RAG1 and RAG2 are both vital for V(D)J recombination. Mice deficient in either RAG1 or RAG2 completely block T and B cell development at progenitor T and B cells stage (Mombaerts et al. 1992; Shinkai et al. 1992). In humans, mutations in RAG1 or RAG2 result in various degrees of residual V(D)J recombination activity, causing a broad spectrum of clinical phenotypes, ranging from severe combined immunodeficiency to autoimmunity (Notarangelo et al. 2016; Delmonte et al. 2018).

Although it is currently not known if and to what extent a reduction in RAG1/2 expression plays a role in the reported decreased antibody response by PFASs, it may be hypothesized that lower RAG1/2 levels in B lymphocytes reduces V(D)J recombination activity, subsequently leading to less diverse repertoire of immunoglobulins. As a consequence, antigens will not be properly recognized by cell surface immunoglobulins, preventing activation of the B lymphocytes and differentiation into plasma cells, producing large amounts of immunoglobulins. Although out of the scope of this study, if PFASs also reduce RAG1/2 expression in T lymphocytes, then T lymphocytes will have less functional T cell receptors (TCRs) for effective recognition of antigens since RAG genes are known to play a crucial role in shaping the TCR repertoire and T cell development (Hosokawa and Rothenberg 2021). This in turn can affect not only the cellular T cell-response but also the T cell-dependent antibody response (TDAR) as insufficiently activated T lymphocytes produce less cytokines, such as IL-2, which are needed to stimulate B lymphocytes to produce immunoglobulins (Komatsu et al. 2021).

In B lymphocytes, expression of RAG is limited to specific early stages of developing B lymphocytes in bone marrow undergoing immunoglobulin rearrangement. It has been reported that RAG expression is increasing at two stages during B cell development. First in progenitor B cells undergoing recombination of the immunoglobulin heavy chain (IgH). Once the IgH is established, a pre-B cell receptor (pre-BCR) will be expressed at the cell surface which, together with IL7R signaling, leads to cessation of RAG expression and inhibits further IgH recombination, while promoting proliferation, survival and differentiation into precursor B lymphocytes. When precursor B cells leave the cell cycle, they become small precursor B cells. Subsequent signals from the pre-B cell receptor in combination with attenuation of IL7R signaling will cause a second wave of RAG expression leading to recombination of the immunoglobulin light chain (IgL). Interestingly, we also found a clear downregulation (2.8-fold) of the expression of *IL7R*, being part of the affected pathways ‘B Cell Development’ and ‘Primary Immunodeficiency Signaling’ by PFOA in the RNA seq analysis of the present study, which may also play a role in the immunotoxic effects of PFASs. Once a functional immunoglobulin is established, immature B cells downregulate RAG expression (Monroe et al. 1999; Lazorchak and Su 2011; Clark et al. 2014; Winkler and Martensson 2018).

As can be deduced from the description above of the physiological regulation of RAG1/RAG2 in B cell development, one would not expect RAG expression in mature B cells. Although the Namalwa cell line is described as a mature B cell lymphoma cell line, we found that *RAG* genes are highly expressed with Ct values ranging from 23 to 26. The Namalwa cell line is derived from a Human Burkitt’s lymphoma, which may be caused by infection of B cells with Epstein–Barr Virus (EBV) (Hutcheson et al. 2021). It has been observed that EBV-infected B lymphocytes, including Namalwa, do express *RAG1* and *RAG2* genes (Kuhn-Hallek et al. 1995). This indicates that Namalwa cells are a useful in vitro tool to study effects of chemicals and drugs on RAG expression but at the same time one must interpret the data with care as it may not totally reflect normal physiology.

The mechanism underlying the decreased expression of *RAG* genes upon treatment of Namalwa cells with PFASs is not clear. It is known that both *RAG1* and *RAG2* are target genes of FOXO1 and that inactivation of FOXO1 through induction of the PI3K-Akt signaling pathway results in decreased expression of the *RAG* genes (Lazorchak et al. 2010; Lazorchak and Su 2011; Benhamou et al. 2018; Peña-Pérez et al. 2022). The hypothesis that the effect of PFASs on RAG expression in Namalwa cells is mediated by FOXO1 is corroborated by the finding that other immunological relevant genes downregulated by PFASs in Namalwa cells are also FOXO1 target genes. Of the 5 downregulated genes in the ‘B cell development’ pathway, besides *RAG1* and *RAG2*, *IL7R* and *CD79B* are known to be FOXO1 targets (Mansson et al. 2012; Peña-Pérez et al. 2022). In the ‘Primary Immunodeficiency signaling’ pathway not only *RAG1*, *RAG2* and *IL7R* were downregulated but also *AICDA* (Activation Induced Cytidine Deaminase) and *IGLL1* for which there is strong evidence that their expression is regulated by FOXO1 (for reviews, see Szydłowski et al. 2014; Cabrera-Ortega et al. 2017). Moreover, in the present study it was found that inhibition of FOXO1 by AS1842856 also reduced expression of *RAG1* and *RAG2* in Namalwa cells, supporting that *RAG* expression in Namalwa cells is regulated by FOXO1.

Since RAG expression is regulated during B cell development, it is of interest to know whether PFASs can accumulate in the bone marrow, being the site where B cell development takes place. Several studies reported that PFASs have been detected in the bones of PFAS-exposed experimental animals. Dietary exposure studies with adult male C57BL/6 mice showed accumulation of labeled PFOS, PFBS and PFOA in bones which was more pronounced in bone marrow than in the calcified bone (Bogdanska et al. 2011, 2014, 2020). Also two studies with human autopsy material showed deposition of PFOA and PFOS in bones (Pérez et al. 2013; Koskela et al. 2017). When comparing the distribution of the two PFASs over the two bone compartments, PFOS showed higher concentrations in the bone marrow than in the trabecular bone, whereas the concentrations of PFOA in both compartments were similar (Koskela et al. 2017). In a study with male C57BL/6 mice exposed to PFOA or PFOS, it was demonstrated that short-term oral exposure (10 days) to PFOA decreased the number of pro/pre-B cells in the bone marrow at a doses of 0.002% (estimated as 3.1 mg/kg bw/day) and 0.02% (estimated as 23.5 mg/kg bw/day). Although a dose of 0.02% reduced food intake by 30%, the lower dose of 0.002% did not affect food intake. PFOS only decreased the number of pro/pre-B cells in the bone marrow at a dose of 0.02% (estimated as 23.5 mg/kg bw/day), a dose which caused a 23% reduction in food intake. These findings suggest a specific effect of PFOA on B cell development at doses that do not cause general toxicity (Qazi et al. 2012). Thus, the outcome of the present work together with results obtained by others on the disposition and effects of PFASs in bone marrow warrants further investigation into the impact of these compounds on immune cells in the bone marrow. Moreover, the data indicate that the bone marrow is a relevant body compartment to consider when setting up PBK models for PFASs.

The EFSA CONTAM Panel concluded that at this stage no in vivo data are available to derive RPFs for effects on the immune system. Novel approach methods, such as the one applied in the current study, may contribute to derivation of such RPFs that are relevant for humans. It is not known to which extent a reduction in *RAG1/2* expression plays a role in the reported decreased antibody response, and whether in vitro RPFs based on *RAG1/2* expression are relevant to be applied in risk assessment. PFASs have been shown to affect various immune cells in vitro, including dendritic cells and B cells (e.g., Ahuja et al. 2009; Brieger et al. 2011; Corsini et al. 2011, 2012; Midgett et al. 2015; Berntsen et al. 2022), and it remains to be established which effects are critical for the decrease in antibody response in vivo, which may also result from the combined effects on targeting different immune cells at the same time. When combining the outcomes of in vitro studies in different immune cells, a more complete picture may be obtained regarding the mechanisms underlying the PFAS-induced decrease in antibody response.

Altogether, the present study shows that PFOA, PFNA, PFOS and PFHxS decrease the expression of *RAG1* and *RAG2* in the human B cell line Namalwa. Such a reduction of *RAG1* and *RAG2* expression may play a role in the reduced antibody response induced by several PFASs and related data may be contribute to derive RPFs that are relevant for humans.

## Supplementary Information

Below is the link to the electronic supplementary material.

Supplementary file1 (DOCX 150 KB)

Supplementary file2 (XLSX 16 KB)

Supplementary file3 (XLSX 24 KB)

## References

[CR251] Abraham K, Mielke H, Fromme H (2020). Internal exposure to perfluoroalkyl substances (PFASs) and biological markers in 101 healthy 1-year-old children: associations between levels of perfluorooctanoic acid (PFOA) and vaccine response. Arch Toxicol.

[CR1] Ahuja V, Eisenblätter M, Ignatius R, Stahlmann R (2009). Ammonium perfluorooctanoate substantially alters phenotype and cytokine secretion of human monocyte-derived dendritic cells in vitro. Immunopharmacol Immunotoxicol.

[CR2] Anders S, Pyl PT, Huber W (2015). HTSeq-A Python framework to work with high-throughput sequencing data. Bioinformatics.

[CR3] ATSDR (2021) Toxicological profile for perfluoroalkyls. version May 2021 https://www.atsdr.cdc.gov/toxprofiles/tp200.pdf. 37220203

[CR4] Bassing CH, Swat W, Alt FW (2002). The mechanism and regulation of chromosomal V(D)J recombination. Cell.

[CR5] Behr AC, Plinsch C, Braeuning A, Buhrke T (2020). Activation of human nuclear receptors by perfluoroalkylated substances (PFAS). Toxicol Vitr.

[CR6] Benhamou D, Labi V, Getahun A (2018). The c-Myc/miR17-92/PTEN axis tunes PI3K activity to control expression of recombination activating genes in early b cell development. Front Immunol.

[CR7] Berntsen HF, Bodin J, Øvrevik J (2022). A human relevant mixture of persistent organic pollutants induces reactive oxygen species formation in isolated human leucocytes: Involvement of the β2-adrenergic receptor. Environ Int.

[CR8] Bogdanska J, Borg D, Sundström M (2011). Tissue distribution of 35S-labelled perfluorooctane sulfonate in adult mice after oral exposure to a low environmentally relevant dose or a high experimental dose. Toxicology.

[CR9] Bogdanska J, Sundström M, Bergström U (2014). Tissue distribution of 35S-labelled perfluorobutanesulfonic acid in adult mice following dietary exposure for 1–5days. Chemosphere.

[CR10] Bogdanska J, Borg D, Bergström U (2020). Tissue distribution of 14C-labelled perfluorooctanoic acid in adult mice after 1–5 days of dietary exposure to an experimental dose or a lower dose that resulted in blood levels similar to those detected in exposed humans. Chemosphere.

[CR11] Brieger A, Bienefeld N, Hasan R (2011). Impact of perfluorooctanesulfonate and perfluorooctanoic acid on human peripheral leukocytes. Toxicol Vitr.

[CR12] Cabrera-Ortega AA, Feinberg D, Liang Y (2017). The role of forkhead box 1 (FOXO1) in the immune system: Dendritic cells, T cells, B cells, and hematopoietic stem cells. Crit Rev Immunol.

[CR13] Chen Y, Lun ATL, Smyth GK (2016). From reads to genes to pathways: differential expression analysis of RNA-Seq experiments using Rsubread and the edgeR quasi-likelihood pipeline. F1000Res.

[CR14] Clark MM, Mandal M, Ochiai K, Sing H (2014). Orchestrating B cell lymphopoiesis through interplay of IL-7 receptor and pre-B cell receptor signalling. Nat Rev Immunol.

[CR15] Corsini E, Avogadro A, Galbiati V (2011). In vitro evaluation of the immunotoxic potential of perfluorinated compounds (PFCs). Toxicol Appl Pharmacol.

[CR16] Corsini E, Sangiovanni E, Avogadro A (2012). In vitro characterization of the immunotoxic potential of several perfluorinated compounds (PFCs). Toxicol Appl Pharmacol.

[CR17] Danecek P, Bonfield JK, Liddle J (2021). Twelve years of SAMtools and BCFtools. Gigascience.

[CR18] Delmonte OM, Schuetz C, Notarangelo LD (2018). RAG deficiency: two genes, many diseases. J Clin Immunol.

[CR19] DeWitt JC, Copeland CB, Strynar MJ, Luebke RW (2008). Perfluorooctanoic acid-induced immunomodulation in adult C57BL/6J or C57BL/6N female mice. Environ Health Perspect.

[CR20] DeWitt JC, Copeland CB, Luebke RW (2009). Suppression of humoral immunity by perfluorooctanoic acid is independent of elevated serum corticosterone concentration in mice. Toxicol Sci.

[CR21] DeWitt JC, Williams WC, Creech NJ, Luebke RW (2016). Suppression of antigen-specific antibody responses in mice exposed to perfluorooctanoic acid: Role of PPAR and T and B cell targeting. J Immunotoxicol.

[CR22] Dong GH, Zhang YH, Zheng L (2009). Chronic effects of perfluorooctanesulfonate exposure on immunotoxicity in adult male C57BL/6 mice. Arch Toxicol.

[CR23] Dong GH, Liu MM, Wang D (2011). Sub-chronic effect of perfluorooctanesulfonate (PFOS) on the balance of type 1 and type 2 cytokine in adult C57BL6 mice. Arch Toxicol.

[CR24] EFSA Contam Panel (2018). Scientific opinion risk to human health related to the presence of perfluorooctane sulfonic acid and perfluorooctanoic acid in food. EFSA J.

[CR25] EFSA Contam Panel (2020). Scientific opinion risk to human health related to the presence of per fluoroalkyl substances in food. EFSA J.

[CR26] Evans N, Conley JM, Cardon M (2022). In vitro activity of a panel of per and polyfluoroalkyl substances (PFAS), fatty acids, and pharmaceuticals in peroxisome proliferator-activated receptor (PPAR) alpha, PPAR gamma, and estrogen receptor assays. Toxicol Appl Pharmacol.

[CR27] Gan T, Wang Y, Liu Y (2021). RAG2 abolishes RAG1 aggregation to facilitate V(D)J recombination. Cell Rep.

[CR28] Hosokawa H, Rothenberg EV (2021). How transcription factors drive choice of the T cell fate. Nat Rev Immunol.

[CR29] Hutcheson RL, Chakravorty A, Sugden B (2021). Burkitt lymphomas evolve to escape dependencies on epstein-barr virus. Front Cell Infect Microbiol.

[CR30] Kim D, Paggi JM, Park C (2019). Graph-based genome alignment and genotyping with HISAT2 and HISAT-genotype. Nat Biotechnol.

[CR31] Komatsu H, Sugimoto J, Goto K (2021). Adverse Outcome Pathway on inhibition of calcineurin activity leading to impaired T-cell dependent antibody response. OECD series on adverse outcome pathways.

[CR32] Koskela A, Koponen J, Lehenkari P (2017). Perfluoroalkyl substances in human bone: concentrations in bones and effects on bone cell differentiation. Sci Rep.

[CR33] Kuhn-Hallek I, Sage DR, Stein L (1995). Expression of recombination activating genes (RAG-l and RAG-2) in Epstein-Barr virus-bearing B cells. Blood.

[CR34] Lazorchak AS, Su B (2011). Perspectives on the role of mTORC2 in B lymphocyte development, immunity and tumorigenesis. Protein Cell.

[CR35] Lazorchak AS, Liu D, Facchinetti V (2010). Sin1-mTORC2 suppresses rag and il7r gene expression through Akt2 in B cells. Mol Cell.

[CR36] Lefebvre DE, Curran I, Armstrong C (2008). Immunomodulatory effects of dietary potassium perfluorooctane sulfonate (PFOS) exposure in adult Sprague-Dawley rats. J Toxicol Environ Health A.

[CR37] Liang L, Pan Y, Bin L (2022). Immunotoxicity mechanisms of perfluorinated compounds PFOA and PFOS. Chemosphere.

[CR38] Mansson R, Welinder E, Åhsberg J (2012). Positive intergenic feedback circuitry, involving EBF1 and FOXO1, orchestrates B-cell fate. Proc Natl Acad Sci USA.

[CR39] Martin M (2011). Cutadapt removes adapter sequences from high-throughput sequencing reads. Embnet J.

[CR40] McCarthy DJ, Chen Y, Smyth GK (2012). Differential expression analysis of multifactor RNA-Seq experiments with respect to biological variation. Nucleic Acids Res.

[CR41] Midgett K, Peden-Adams MM, Gilkeson GS, Kamen DL (2015). In vitro evaluation of the effects of perfluorooctanesulfonic acid (PFOS) and perfluorooctanoic acid (PFOA) on IL-2 production in human T-cells. J Appl Toxicol.

[CR42] Mo X, Bailin T, Sadofsky MJ (1999). RAG1 and RAG2 cooperate in specific binding to the recombination signal sequence in vitro. J Biol Chem.

[CR43] Mombaerts P, Iacomini J, Johnson RS (1992). RAG-1-Deficient Mice Have No Mature B and T Lymphocytes. Cell.

[CR44] Monroe RJ, Chen F, Ferrini R (1999). RAG2 is regulated differentially in B and T cells by elements 5′ of the promoter. Proc Natl Acad Sci USA.

[CR45] Notarangelo LD, Kim M-S, Walter JE, Lee YN (2016). Human RAG mutations: biochemistry and clinical implications. Nat Rev Immunol.

[CR46] Peden-Adams MM, Keller JM, Eudaly JG (2008). Suppression of humoral immunity in mice following exposure to perfluorooctane sulfonate. Toxicol Sci.

[CR47] Peña-Pérez L, Kharazi S, Frengen N (2022). FOXO Dictates Initiation of B Cell development and myeloid restriction in common lymphoid progenitors. Front Immunol.

[CR48] Pérez F, Nadal M, Navarro-Ortega A (2013). Accumulation of perfluoroalkyl substances in human tissues. Environ Int.

[CR49] Qazi MR, Nelson BD, DePierre JW, Abedi-Valugerdi M (2010). 28-Day dietary exposure of mice to a low total dose (7 mg/kg) of perfluorooctanesulfonate (PFOS) alters neither the cellular compositions of the thymus and spleen nor humoral immune responses: does the route of administration play a pivotal role in PFOS-induced immunotoxicity?. Toxicology.

[CR50] Qazi MR, Dean Nelson B, DePierre JW, Abedi-Valugerdi M (2012). High-dose dietary exposure of mice to perfluorooctanoate or perfluorooctane sulfonate exerts toxic effects on myeloid and B-lymphoid cells in the bone marrow and these effects are partially dependent on reduced food consumption. Food Chem Toxicol.

[CR51] Ritchie ME, Phipson B, Wu D (2015). Limma powers differential expression analyses for RNA-sequencing and microarray studies. Nucleic Acids Res.

[CR52] Robinson MD, McCarthy DJ, Smyth GK (2009). edgeR: a bioconductor package for differential expression analysis of digital gene expression data. Bioinformatics.

[CR53] Schatz DG, Ji Y (2011). Recombination centres and the orchestration of V(D)J recombination. Nat Rev Immunol.

[CR54] Schmieder R, Edwards R (2011). Quality control and preprocessing of metagenomic datasets. Bioinformatics.

[CR55] Shinkai Y, Gary R, Lam KP (1992). RAG-2-deficient mice lack mature lymphocytes owing to inability to initiate V(D)J rearrangement. Cell.

[CR56] Szydłowski M, Jabłońska E, Juszczyński P (2014). FOXO1 Transcription Factor: a critical effector of the PI3K-AKT axis in B-cell development. Int Rev Immunol.

[CR57] Van Belle K, Herman J, Boon L (2016). Comparative in vitro immune stimulation analysis of primary human B cells and B cell lines. J Immunol Res.

[CR58] Vetvicka V, Vetvickova J (2013). Reversal of perfluorooctanesulfonate-induced immunotoxicity by a glucan-resveratrol-vitamin C combination. Orient Pharm Exp Med.

[CR59] Wang L, Wang S, Li W (2012). RSeQC: Quality control of RNA-seq experiments. Bioinformatics.

[CR60] Wang Z, Dewitt JC, Higgins CP, Cousins IT (2017). A never-ending story of Per- and Polyfluoroalkyl Substances (PFASs)?. Environ Sci Technol.

[CR61] Winkler TH, Martensson IL (2018). The role of the pre-b cell receptor in b cell development, repertoire selection, and tolerance. Front Immunol.

[CR62] Zheng L, Dong GH, Jin YH, He QC (2009). Immunotoxic changes associated with a 7-day oral exposure to perfluorooctanesulfonate (PFOS) in adult male C57BL/6 mice. Arch Toxicol.

[CR63] Zheng L, Dong GH, Zhang YH (2011). Type 1 and Type 2 cytokines imbalance in adult male C57BL/6 mice following a 7-day oral exposure to perfluorooctanesulfonate (PFOS). J Immunotoxicol.

