# Supplementary material for: Perfluoroalkyl substances (PFASs) decrease the expression of recombination-activating genes (RAG1 and RAG2) in human B lymphoma Namalwa cells
Source: Arch Toxicol. 2022 Nov 3;97(2):457–68. doi: 10.1007/s00204-022-03405-z (PMC9859925; doi:10.1007/s00204-022-03405-z)
Supplement: Supplementary file 1 — Supplementary file1 (DOCX 150 KB) [file 204_2022_3405_MOESM1_ESM.docx]

**Supplementary data for:**

**Perfluoroalkyl substances (PFASs) decrease the expression of recombination-activating genes (*RAG1* and *RAG2*) in human B lymphoma Namalwa cells**

Aafke WF Janssen^1*^, Jochem Louisse^1^, Deborah Rijkers^1^, Nicole ET Pinckaers^1^, Sjoerdtje A Hoekstra^1^, Ron LAP Hoogenboom^1^, Ad ACM Peijnenburg^1^, Karsten Beekmann^1^

^1^Wageningen Food Safety Research (WFSR), Wageningen University and Research, Akkermaalsbos 2, 6708 WB Wageningen, The Netherlands

***Corresponding author:**

Aafke W.F. Janssen PhD

Wageningen Food Safety Research

Akkermaalsbos 2

6708 WB Wageningen

The Netherlands

Phone: +31 317 483670

Email: aafke.janssen@wur.nl

*Supplementary Table 1. Suppliers, purities, catalog numbers, CAS numbers and maximum concentrations of chemicals tested in the present study.*

| **Chemical** | **Full name** | **Supplier** | **Purity** | **Catalog number** | **CAS number** | **Highest tested concentration (µM)** |
| --- | --- | --- | --- | --- | --- | --- |
| PFOA | Perfluorooctanoic acid | Sigma-Aldrich | 95% | 171468-5G | 335-67-1 | 100 |
| PFNA | Perfluorononanoic acid | Sigma-Aldrich | 99% | 91977-50MG | 375-95-1 | 100 |
| PFHxS | Perfluorohexane sulfonate | Synquest laboratories | 95% | 6164-3-2T | 355-46-4 | 100 |
| PFOS | Perfluorooctane sulfonate | Synquest laboratories | 97% | 6164-3-08 | 1763-23-1 | 100 |
| AS1842856 | - | Sigma-Aldrich | - | 344355-10MG | - | 0.1 |

*Supplementary Table 2. Differentially expressed genes in Namalwa cells exposed to PFOA for 48 h ranked by p-value (based on a statistical cut-off of P<0.01 and 2log fold change of >0.5).*

| **Ensembl Gene ID** | **Fold change** | **P-value** | **Gene Symbol** |
| --- | --- | --- | --- |
| ENSG00000166349 | -4.68 | 2.436E-15 | RAG1 |
| ENSG00000211459 | 2.57 | 6.639E-14 | MT-RNR1 |
| ENSG00000175097 | -3.47 | 5.527E-13 | RAG2 |
| ENSG00000076641 | 1.69 | 7.230E-12 | PAG1 |
| ENSG00000100721 | -1.75 | 8.121E-12 | TCL1A |
| ENSG00000235513 | 4.91 | 1.328E-11 | L3MBTL2-AS1 |
| ENSG00000134548 | 2.73 | 1.638E-11 | SPX |
| ENSG00000180573 | 1.93 | 2.788E-11 | H2AC6 |
| ENSG00000157514 | 1.80 | 3.147E-11 | TSC22D3 |
| ENSG00000072274 | -1.60 | 3.451E-11 | TFRC |
| ENSG00000111732 | -1.67 | 6.166E-11 | AICDA |
| ENSG00000117318 | -2.05 | 7.829E-11 | ID3 |
| ENSG00000196230 | -1.52 | 8.547E-11 | TUBB |
| ENSG00000198796 | 2.53 | 8.806E-11 | ALPK2 |
| ENSG00000146670 | -1.82 | 1.525E-10 | CDCA5 |
| ENSG00000089280 | -1.53 | 1.660E-10 | FUS |
| ENSG00000135069 | 1.50 | 2.368E-10 | PSAT1 |
| ENSG00000123416 | -1.50 | 2.444E-10 | TUBA1B |
| ENSG00000237649 | -1.66 | 4.154E-10 | KIFC1 |
| ENSG00000142945 | -1.60 | 4.476E-10 | KIF2C |
| ENSG00000089685 | -1.57 | 5.705E-10 | BIRC5 |
| ENSG00000074800 | -1.49 | 6.928E-10 | ENO1 |
| ENSG00000135476 | -1.66 | 8.666E-10 | ESPL1 |
| ENSG00000257275 | -2.24 | 1.002E-09 | ENSG00000257275 |
| ENSG00000111206 | -1.58 | 1.056E-09 | FOXM1 |
| ENSG00000142765 | 1.70 | 1.060E-09 | SYTL1 |
| ENSG00000164104 | -1.50 | 1.074E-09 | HMGB2 |
| ENSG00000101255 | 1.53 | 1.117E-09 | TRIB3 |
| ENSG00000090975 | -1.84 | 1.152E-09 | PITPNM2 |
| ENSG00000127325 | -2.09 | 1.351E-09 | BEST3 |
| ENSG00000162408 | -1.55 | 2.355E-09 | NOL9 |
| ENSG00000276043 | -1.74 | 2.707E-09 | UHRF1 |
| ENSG00000188229 | -1.52 | 2.814E-09 | TUBB4B |
| ENSG00000134333 | -1.73 | 3.141E-09 | LDHA |
| ENSG00000121966 | -1.49 | 3.232E-09 | CXCR4 |
| ENSG00000007968 | -1.65 | 3.256E-09 | E2F2 |
| ENSG00000100413 | -1.69 | 3.823E-09 | POLR3H |
| ENSG00000101224 | -1.53 | 3.829E-09 | CDC25B |
| ENSG00000079616 | -1.55 | 4.046E-09 | KIF22 |
| ENSG00000089041 | 1.64 | 4.156E-09 | P2RX7 |
| ENSG00000131747 | -1.50 | 4.316E-09 | TOP2A |
| ENSG00000189403 | -1.46 | 4.687E-09 | HMGB1 |
| ENSG00000254709 | -1.59 | 5.013E-09 | IGLL5 |
| ENSG00000066279 | -1.53 | 5.417E-09 | ASPM |
| ENSG00000178999 | -1.75 | 5.507E-09 | AURKB |
| ENSG00000173207 | -1.44 | 5.612E-09 | CKS1B |
| ENSG00000135842 | 1.66 | 5.642E-09 | NIBAN1 |
| ENSG00000198380 | 1.54 | 5.893E-09 | GFPT1 |
| ENSG00000111665 | -1.78 | 6.023E-09 | CDCA3 |
| ENSG00000170312 | -1.57 | 6.097E-09 | CDK1 |
| ENSG00000105011 | -1.62 | 6.119E-09 | ASF1B |
| ENSG00000113369 | 1.67 | 6.732E-09 | ARRDC3 |
| ENSG00000175567 | -1.47 | 8.265E-09 | UCP2 |
| ENSG00000182481 | -1.46 | 9.588E-09 | KPNA2 |
| ENSG00000109971 | -1.45 | 1.014E-08 | HSPA8 |
| ENSG00000171848 | -1.62 | 1.016E-08 | RRM2 |
| ENSG00000103522 | 1.53 | 1.183E-08 | IL21R |
| ENSG00000170909 | 2.50 | 1.214E-08 | OSCAR |
| ENSG00000102445 | -1.45 | 1.229E-08 | RUBCNL |
| ENSG00000149503 | -1.54 | 1.233E-08 | INCENP |
| ENSG00000109805 | -1.51 | 1.253E-08 | NCAPG |
| ENSG00000134690 | -1.46 | 1.333E-08 | CDCA8 |
| ENSG00000187837 | 2.14 | 1.477E-08 | H1-2 |
| ENSG00000198805 | -1.48 | 1.668E-08 | PNP |
| ENSG00000166750 | 1.50 | 1.921E-08 | SLFN5 |
| ENSG00000166482 | 1.87 | 2.268E-08 | MFAP4 |
| ENSG00000189423 | 2.76 | 2.512E-08 | USP32P3 |
| ENSG00000075218 | -1.65 | 2.586E-08 | GTSE1 |
| ENSG00000128965 | 1.56 | 2.645E-08 | CHAC1 |
| ENSG00000158079 | 3.67 | 2.651E-08 | PTPDC1 |
| ENSG00000007312 | -1.44 | 2.956E-08 | CD79B |
| ENSG00000187741 | -1.66 | 2.967E-08 | FANCA |
| ENSG00000136997 | -1.48 | 3.227E-08 | MYC |
| ENSG00000169435 | -1.50 | 3.411E-08 | RASSF6 |
| ENSG00000110090 | 1.85 | 3.417E-08 | CPT1A |
| ENSG00000114346 | -1.51 | 3.812E-08 | ECT2 |
| ENSG00000164611 | -1.46 | 3.919E-08 | PTTG1 |
| ENSG00000033327 | 1.59 | 4.288E-08 | GAB2 |
| ENSG00000171314 | -1.45 | 5.393E-08 | PGAM1 |
| ENSG00000128322 | -2.06 | 5.521E-08 | IGLL1 |
| ENSG00000180198 | -1.48 | 5.921E-08 | RCC1 |
| ENSG00000189057 | -1.58 | 5.960E-08 | FAM111B |
| ENSG00000144619 | 4.28 | 6.008E-08 | CNTN4 |
| ENSG00000132967 | -1.55 | 6.163E-08 | HMGB1P5 |
| ENSG00000065328 | -1.62 | 6.250E-08 | MCM10 |
| ENSG00000169570 | 2.22 | 6.327E-08 | DTWD2 |
| ENSG00000145386 | -1.49 | 6.882E-08 | CCNA2 |
| ENSG00000116852 | 1.55 | 6.971E-08 | KIF21B |
| ENSG00000128165 | 1.78 | 7.207E-08 | ADM2 |
| ENSG00000115977 | 1.60 | 7.572E-08 | AAK1 |
| ENSG00000140848 | -1.58 | 7.950E-08 | CPNE2 |
| ENSG00000151012 | 1.54 | 9.195E-08 | SLC7A11 |
| ENSG00000087074 | 1.75 | 9.739E-08 | PPP1R15A |
| ENSG00000135451 | -1.54 | 9.770E-08 | TROAP |
| ENSG00000161888 | -1.62 | 1.019E-07 | SPC24 |
| ENSG00000204859 | -1.54 | 1.035E-07 | ZBTB48 |
| ENSG00000087266 | 1.51 | 1.094E-07 | SH3BP2 |
| ENSG00000085840 | -1.61 | 1.168E-07 | ORC1 |
| ENSG00000132646 | -1.44 | 1.217E-07 | PCNA |
| ENSG00000162512 | 1.87 | 1.270E-07 | SDC3 |
| ENSG00000123485 | -1.48 | 1.272E-07 | HJURP |
| ENSG00000135540 | 1.88 | 1.303E-07 | NHSL1 |
| ENSG00000138160 | -1.42 | 1.333E-07 | KIF11 |
| ENSG00000228716 | -1.47 | 1.335E-07 | DHFR |
| ENSG00000121390 | -1.44 | 1.481E-07 | PSPC1 |
| ENSG00000121152 | -1.49 | 1.803E-07 | NCAPH |
| ENSG00000164109 | -1.42 | 1.819E-07 | MAD2L1 |
| ENSG00000112576 | -1.48 | 1.831E-07 | CCND3 |
| ENSG00000213347 | -1.77 | 1.912E-07 | MXD3 |
| ENSG00000100162 | -1.60 | 1.993E-07 | CENPM |
| ENSG00000156970 | -1.44 | 2.032E-07 | BUB1B |
| ENSG00000168685 | -2.82 | 2.045E-07 | IL7R |
| ENSG00000221963 | 1.42 | 2.181E-07 | APOL6 |
| ENSG00000139269 | 1.45 | 2.422E-07 | INHBE |
| ENSG00000129173 | -1.57 | 2.669E-07 | E2F8 |
| ENSG00000186185 | -1.58 | 2.986E-07 | KIF18B |
| ENSG00000175063 | -1.53 | 2.999E-07 | UBE2C |
| ENSG00000103489 | 1.47 | 3.008E-07 | XYLT1 |
| ENSG00000104921 | 1.44 | 3.096E-07 | FCER2 |
| ENSG00000114023 | -1.44 | 3.130E-07 | FAM162A |
| ENSG00000169359 | 1.62 | 3.251E-07 | SLC33A1 |
| ENSG00000070669 | 1.77 | 3.373E-07 | ASNS |
| ENSG00000164687 | -1.56 | 3.709E-07 | FABP5 |
| ENSG00000118193 | -1.49 | 3.759E-07 | KIF14 |
| ENSG00000144395 | -1.84 | 3.830E-07 | CCDC150 |
| ENSG00000105928 | 1.94 | 4.212E-07 | GSDME |
| ENSG00000011426 | -1.51 | 4.426E-07 | ANLN |
| ENSG00000165507 | -1.73 | 5.178E-07 | DEPP1 |
| ENSG00000117322 | 1.66 | 6.084E-07 | CR2 |
| ENSG00000162772 | 1.76 | 6.120E-07 | ATF3 |
| ENSG00000197872 | 1.47 | 6.130E-07 | CYRIA |
| ENSG00000080986 | -1.44 | 6.238E-07 | NDC80 |
| ENSG00000070540 | 1.51 | 6.292E-07 | WIPI1 |
| ENSG00000138778 | -1.50 | 6.730E-07 | CENPE |
| ENSG00000162413 | -1.51 | 6.823E-07 | KLHL21 |
| ENSG00000198938 | -1.45 | 7.005E-07 | MT-CO3 |
| ENSG00000150995 | 1.43 | 7.364E-07 | ITPR1 |
| ENSG00000184678 | 1.70 | 7.705E-07 | H2BC21 |
| ENSG00000170006 | 1.49 | 7.930E-07 | TMEM154 |
| ENSG00000148773 | -1.50 | 8.253E-07 | MKI67 |
| ENSG00000133069 | -1.63 | 8.262E-07 | TMCC2 |
| ENSG00000106268 | -1.54 | 8.406E-07 | NUDT1 |
| ENSG00000166451 | -1.46 | 8.407E-07 | CENPN |
| ENSG00000112984 | -1.42 | 8.409E-07 | KIF20A |
| ENSG00000167900 | -1.53 | 8.549E-07 | TK1 |
| ENSG00000138658 | -1.54 | 8.667E-07 | ZGRF1 |
| ENSG00000260916 | 1.90 | 9.388E-07 | CCPG1 |
| ENSG00000122224 | 1.44 | 9.433E-07 | LY9 |
| ENSG00000198467 | -2.26 | 9.802E-07 | TPM2 |
| ENSG00000049249 | 2.16 | 9.895E-07 | TNFRSF9 |
| ENSG00000112742 | -1.49 | 1.022E-06 | TTK |
| ENSG00000128203 | 1.59 | 1.089E-06 | ASPHD2 |
| ENSG00000101447 | -1.81 | 1.112E-06 | FAM83D |
| ENSG00000117877 | -1.54 | 1.125E-06 | POLR1G |
| ENSG00000215417 | -1.88 | 1.139E-06 | MIR17HG |
| ENSG00000130513 | 3.61 | 1.209E-06 | GDF15 |
| ENSG00000165480 | -1.69 | 1.310E-06 | SKA3 |
| ENSG00000075702 | -1.53 | 1.372E-06 | WDR62 |
| ENSG00000122966 | -1.43 | 1.390E-06 | CIT |
| ENSG00000175197 | 1.51 | 1.455E-06 | DDIT3 |
| ENSG00000142731 | -1.57 | 1.469E-06 | PLK4 |
| ENSG00000256663 | -1.89 | 1.498E-06 | ENSG00000256663 |
| ENSG00000188610 | -1.43 | 1.537E-06 | FAM72B |
| ENSG00000198712 | -1.43 | 1.800E-06 | MT-CO2 |
| ENSG00000093009 | -1.57 | 1.883E-06 | CDC45 |
| ENSG00000215492 | -1.41 | 1.917E-06 | HNRNPA1P7 |
| ENSG00000102393 | -1.61 | 2.007E-06 | GLA |
| ENSG00000123384 | 1.55 | 2.177E-06 | LRP1 |
| ENSG00000203760 | -1.72 | 2.258E-06 | CENPW |
| ENSG00000047365 | 1.43 | 2.484E-06 | ARAP2 |
| ENSG00000008710 | 1.57 | 2.632E-06 | PKD1 |
| ENSG00000105514 | 1.53 | 2.872E-06 | RAB3D |
| ENSG00000204054 | 1.87 | 2.877E-06 | LINC00963 |
| ENSG00000138346 | -1.42 | 2.915E-06 | DNA2 |
| ENSG00000104147 | -1.78 | 2.969E-06 | OIP5 |
| ENSG00000112029 | -1.44 | 3.003E-06 | FBXO5 |
| ENSG00000230333 | -1.60 | 3.072E-06 | ENSG00000230333 |
| ENSG00000261609 | 1.63 | 3.166E-06 | GAN |
| ENSG00000244405 | 2.43 | 3.452E-06 | ETV5 |
| ENSG00000186104 | 1.42 | 3.753E-06 | CYP2R1 |
| ENSG00000229191 | 1.68 | 4.063E-06 | ENSG00000229191 |
| ENSG00000114480 | -1.51 | 4.081E-06 | GBE1 |
| ENSG00000200087 | 3.23 | 4.168E-06 | SNORA73B |
| ENSG00000085999 | -1.45 | 4.182E-06 | RAD54L |
| ENSG00000105246 | 1.68 | 4.254E-06 | EBI3 |
| ENSG00000173662 | -2.59 | 4.318E-06 | TAS1R1 |
| ENSG00000259781 | -1.54 | 4.434E-06 | HMGB1P6 |
| ENSG00000198001 | 1.42 | 4.568E-06 | IRAK4 |
| ENSG00000140534 | -1.48 | 4.656E-06 | TICRR |
| ENSG00000138182 | -1.43 | 4.835E-06 | KIF20B |
| ENSG00000143367 | 1.77 | 4.867E-06 | TUFT1 |
| ENSG00000134070 | 2.21 | 5.198E-06 | IRAK2 |
| ENSG00000166171 | -1.54 | 5.383E-06 | DPCD |
| ENSG00000136603 | 1.44 | 5.557E-06 | SKIL |
| ENSG00000196632 | 4.99 | 6.264E-06 | WNK3 |
| ENSG00000163808 | -1.43 | 6.507E-06 | KIF15 |
| ENSG00000172009 | -1.45 | 6.508E-06 | THOP1 |
| ENSG00000111247 | -1.45 | 6.691E-06 | RAD51AP1 |
| ENSG00000003249 | 1.75 | 6.773E-06 | DBNDD1 |
| ENSG00000196550 | -1.58 | 6.866E-06 | FAM72A |
| ENSG00000235760 | 2.05 | 7.227E-06 | MSH2-OT1 |
| ENSG00000127564 | -1.59 | 7.816E-06 | PKMYT1 |
| ENSG00000177311 | 1.47 | 7.932E-06 | ZBTB38 |
| ENSG00000127074 | -1.46 | 8.025E-06 | RGS13 |
| ENSG00000197299 | -1.45 | 8.081E-06 | BLM |
| ENSG00000008517 | 1.58 | 8.168E-06 | IL32 |
| ENSG00000005448 | -1.50 | 8.363E-06 | WDR54 |
| ENSG00000140854 | -1.42 | 8.461E-06 | KATNB1 |
| ENSG00000116183 | 5.97 | 8.498E-06 | PAPPA2 |
| ENSG00000152455 | -1.52 | 8.787E-06 | SUV39H2 |
| ENSG00000261716 | 1.42 | 8.845E-06 | H2BC20P |
| ENSG00000065970 | 1.52 | 9.785E-06 | FOXJ2 |
| ENSG00000154898 | 2.72 | 9.825E-06 | CCDC144CP |
| ENSG00000121621 | -1.44 | 1.063E-05 | KIF18A |
| ENSG00000158406 | 1.64 | 1.088E-05 | H4C8 |
| ENSG00000161791 | 1.54 | 1.175E-05 | FMNL3 |
| ENSG00000134222 | -1.47 | 1.191E-05 | PSRC1 |
| ENSG00000081377 | 1.97 | 1.198E-05 | CDC14B |
| ENSG00000127589 | -1.45 | 1.214E-05 | TUBBP1 |
| ENSG00000169403 | 1.54 | 1.231E-05 | PTAFR |
| ENSG00000137404 | -1.44 | 1.302E-05 | NRM |
| ENSG00000158402 | -1.62 | 1.310E-05 | CDC25C |
| ENSG00000263934 | 2.33 | 1.451E-05 | SNORD3A |
| ENSG00000169442 | -1.47 | 1.478E-05 | CD52 |
| ENSG00000186871 | -1.60 | 1.513E-05 | ERCC6L |
| ENSG00000102962 | 1.52 | 1.574E-05 | CCL22 |
| ENSG00000268119 | -1.68 | 1.585E-05 | ENSG00000268119 |
| ENSG00000134352 | 1.43 | 1.630E-05 | IL6ST |
| ENSG00000198855 | 1.52 | 1.925E-05 | FICD |
| ENSG00000215784 | -1.51 | 1.973E-05 | FAM72D |
| ENSG00000151365 | -1.71 | 2.049E-05 | THRSP |
| ENSG00000026103 | 1.46 | 2.059E-05 | FAS |
| ENSG00000055118 | -1.44 | 2.089E-05 | KCNH2 |
| ENSG00000133687 | 5.63 | 2.118E-05 | TMTC1 |
| ENSG00000171241 | -1.41 | 2.162E-05 | SHCBP1 |
| ENSG00000175782 | -1.44 | 2.189E-05 | SLC35E3 |
| ENSG00000231424 | -1.49 | 2.260E-05 | ENSG00000231424 |
| ENSG00000254838 | 1.58 | 2.324E-05 | GVINP1 |
| ENSG00000069974 | 1.54 | 2.501E-05 | RAB27A |
| ENSG00000168078 | -1.56 | 2.616E-05 | PBK |
| ENSG00000051341 | -1.41 | 2.643E-05 | POLQ |
| ENSG00000117394 | -1.42 | 2.673E-05 | SLC2A1 |
| ENSG00000165891 | -1.49 | 2.732E-05 | E2F7 |
| ENSG00000267123 | -4.64 | 2.872E-05 | SCAT1 |
| ENSG00000185278 | 1.42 | 2.880E-05 | ZBTB37 |
| ENSG00000051180 | -1.51 | 2.912E-05 | RAD51 |
| ENSG00000083937 | 1.55 | 2.931E-05 | CHMP2B |
| ENSG00000130487 | 2.30 | 3.123E-05 | KLHDC7B |
| ENSG00000173334 | -1.42 | 3.175E-05 | TRIB1 |
| ENSG00000091073 | 1.45 | 3.331E-05 | DTX2 |
| ENSG00000152766 | 5.92 | 3.359E-05 | ANKRD22 |
| ENSG00000186862 | 1.58 | 3.424E-05 | PDZD7 |
| ENSG00000114554 | 1.43 | 3.608E-05 | PLXNA1 |
| ENSG00000158373 | 1.66 | 3.750E-05 | H2BC5 |
| ENSG00000273018 | 2.40 | 3.767E-05 | FAM106A |
| ENSG00000010932 | -1.55 | 4.020E-05 | FMO1 |
| ENSG00000289194 | -1.79 | 4.056E-05 | ENSG00000289194 |
| ENSG00000230825 | 2.68 | 4.702E-05 | ENSG00000230825 |
| ENSG00000168393 | -1.45 | 4.706E-05 | DTYMK |
| ENSG00000174371 | -1.42 | 4.723E-05 | EXO1 |
| ENSG00000101076 | 5.49 | 4.966E-05 | HNF4A |
| ENSG00000211649 | -1.72 | 4.977E-05 | IGLV7-46 |
| ENSG00000248932 | 1.53 | 5.099E-05 | COPB2-DT |
| ENSG00000131389 | -1.42 | 5.282E-05 | SLC6A6 |
| ENSG00000263006 | 1.67 | 5.377E-05 | ROCK1P1 |
| ENSG00000104413 | 2.22 | 5.498E-05 | ESRP1 |
| ENSG00000152253 | -1.44 | 5.563E-05 | SPC25 |
| ENSG00000166068 | 1.42 | 5.571E-05 | SPRED1 |
| ENSG00000078589 | -1.45 | 5.656E-05 | P2RY10 |
| ENSG00000000460 | -1.45 | 5.715E-05 | C1orf112 |
| ENSG00000132837 | 2.50 | 5.728E-05 | DMGDH |
| ENSG00000237541 | 1.57 | 5.741E-05 | HLA-DQA2 |
| ENSG00000166262 | 1.60 | 5.984E-05 | FAM227B |
| ENSG00000227398 | 1.82 | 6.170E-05 | KIF9-AS1 |
| ENSG00000160957 | -1.45 | 6.571E-05 | RECQL4 |
| ENSG00000251192 | 1.56 | 6.649E-05 | ZNF674 |
| ENSG00000198727 | -1.43 | 6.796E-05 | MT-CYB |
| ENSG00000186162 | 1.58 | 7.127E-05 | CIDECP1 |
| ENSG00000183307 | 1.42 | 7.331E-05 | TMEM121B |
| ENSG00000019549 | -1.87 | 7.636E-05 | SNAI2 |
| ENSG00000276168 | 1.91 | 7.802E-05 | RN7SL1 |
| ENSG00000131351 | -1.47 | 7.839E-05 | HAUS8 |
| ENSG00000167103 | 2.02 | 8.432E-05 | PIP5KL1 |
| ENSG00000035499 | -1.49 | 8.856E-05 | DEPDC1B |
| ENSG00000263513 | -1.52 | 9.341E-05 | FAM72C |
| ENSG00000182010 | -1.44 | 1.033E-04 | RTKN2 |
| ENSG00000284946 | -2.07 | 1.057E-04 | ENSG00000284946 |
| ENSG00000144120 | -1.43 | 1.077E-04 | TMEM177 |
| ENSG00000104356 | -1.43 | 1.160E-04 | POP1 |
| ENSG00000135363 | 1.62 | 1.164E-04 | LMO2 |
| ENSG00000042493 | 1.42 | 1.175E-04 | CAPG |
| ENSG00000124575 | 2.67 | 1.187E-04 | H1-3 |
| ENSG00000165501 | -1.52 | 1.197E-04 | LRR1 |
| ENSG00000145476 | 1.62 | 1.243E-04 | CYP4V2 |
| ENSG00000253626 | -1.54 | 1.309E-04 | EIF5AL1 |
| ENSG00000168679 | 4.06 | 1.339E-04 | SLC16A4 |
| ENSG00000109881 | -1.48 | 1.347E-04 | CCDC34 |
| ENSG00000210100 | 2.52 | 1.377E-04 | MT-TI |
| ENSG00000248599 | 2.84 | 1.390E-04 | FLJ42969 |
| ENSG00000214223 | -1.65 | 1.403E-04 | HNRNPA1P10 |
| ENSG00000197629 | -1.47 | 1.423E-04 | MPEG1 |
| ENSG00000259520 | 3.00 | 1.488E-04 | SLC28A2-AS1 |
| ENSG00000101945 | -1.43 | 1.518E-04 | SUV39H1 |
| ENSG00000197714 | 1.93 | 1.524E-04 | ZNF460 |
| ENSG00000231607 | -1.44 | 1.578E-04 | DLEU2 |
| ENSG00000239665 | 1.47 | 1.583E-04 | ENSG00000239665 |
| ENSG00000251141 | 1.70 | 1.618E-04 | MRPS30-DT |
| ENSG00000143942 | -1.59 | 1.667E-04 | CHAC2 |
| ENSG00000122884 | -1.49 | 1.689E-04 | P4HA1 |
| ENSG00000129048 | -1.75 | 1.692E-04 | ACKR4 |
| ENSG00000181577 | 1.42 | 1.866E-04 | C6orf223 |
| ENSG00000240303 | 1.71 | 1.885E-04 | ACAD11 |
| ENSG00000283696 | -1.63 | 1.911E-04 | ENSG00000283696 |
| ENSG00000112195 | 1.62 | 1.972E-04 | TREML2 |
| ENSG00000253608 | -3.33 | 1.988E-04 | ENSG00000253608 |
| ENSG00000187621 | -1.75 | 2.023E-04 | TCL6 |
| ENSG00000197860 | 1.42 | 2.025E-04 | SGTB |
| ENSG00000164087 | -1.46 | 2.161E-04 | POC1A |
| ENSG00000101187 | -1.65 | 2.248E-04 | SLCO4A1 |
| ENSG00000264112 | 1.97 | 2.296E-04 | ENSG00000264112 |
| ENSG00000175489 | 2.16 | 2.315E-04 | LRRC25 |
| ENSG00000267278 | 1.56 | 2.556E-04 | MAP3K14-AS1 |
| ENSG00000289474 | 3.02 | 2.619E-04 | ENSG00000289474 |
| ENSG00000071539 | -1.43 | 2.705E-04 | TRIP13 |
| ENSG00000176595 | 1.81 | 2.708E-04 | KBTBD11 |
| ENSG00000211663 | -1.45 | 2.761E-04 | IGLV3-19 |
| ENSG00000176171 | -1.71 | 2.776E-04 | BNIP3 |
| ENSG00000157734 | -1.46 | 2.836E-04 | SNX22 |
| ENSG00000092871 | 1.47 | 2.927E-04 | RFFL |
| ENSG00000148835 | -1.42 | 2.927E-04 | TAF5 |
| ENSG00000214331 | -1.60 | 2.958E-04 | PDPR2P |
| ENSG00000167995 | 1.58 | 3.025E-04 | BEST1 |
| ENSG00000014257 | -1.78 | 3.238E-04 | ACP3 |
| ENSG00000289120 | -1.88 | 3.436E-04 | ENSG00000289120 |
| ENSG00000165030 | 1.43 | 3.558E-04 | NFIL3 |
| ENSG00000005844 | 1.74 | 3.667E-04 | ITGAL |
| ENSG00000073905 | -1.66 | 3.764E-04 | VDAC1P1 |
| ENSG00000130635 | -3.05 | 4.032E-04 | COL5A1 |
| ENSG00000140939 | 1.42 | 4.038E-04 | NOL3 |
| ENSG00000166548 | 1.67 | 4.081E-04 | TK2 |
| ENSG00000138587 | -1.49 | 4.160E-04 | MNS1 |
| ENSG00000136052 | 1.61 | 4.251E-04 | SLC41A2 |
| ENSG00000119771 | 1.46 | 4.434E-04 | KLHL29 |
| ENSG00000169575 | -2.56 | 4.713E-04 | VPREB1 |
| ENSG00000162062 | -1.51 | 4.815E-04 | TEDC2 |
| ENSG00000160062 | 1.67 | 4.838E-04 | ZBTB8A |
| ENSG00000279414 | 2.11 | 4.938E-04 | CDRT15P9 |
| ENSG00000275464 | -1.62 | 5.060E-04 | ENSG00000275464 |
| ENSG00000101746 | -1.68 | 5.142E-04 | NOL4 |
| ENSG00000138835 | 1.51 | 5.159E-04 | RGS3 |
| ENSG00000269416 | -5.93 | 5.276E-04 | LINC01224 |
| ENSG00000163297 | 2.07 | 5.538E-04 | ANTXR2 |
| ENSG00000110492 | 1.69 | 5.579E-04 | MDK |
| ENSG00000164920 | -1.61 | 5.809E-04 | OSR2 |
| ENSG00000197099 | 3.48 | 5.910E-04 | ENSG00000197099 |
| ENSG00000248008 | -1.43 | 5.969E-04 | NRAV |
| ENSG00000185275 | -1.93 | 6.109E-04 | CD24P4 |
| ENSG00000154175 | 3.53 | 6.182E-04 | ABI3BP |
| ENSG00000188707 | -1.59 | 6.205E-04 | ZBED6CL |
| ENSG00000001630 | 1.46 | 6.206E-04 | CYP51A1 |
| ENSG00000096968 | 1.43 | 6.227E-04 | JAK2 |
| ENSG00000168061 | -1.55 | 6.234E-04 | SAC3D1 |
| ENSG00000253140 | -1.45 | 6.282E-04 | ENSG00000253140 |
| ENSG00000203814 | 2.45 | 6.411E-04 | H2BC18 |
| ENSG00000186638 | -1.43 | 6.637E-04 | KIF24 |
| ENSG00000172575 | 1.64 | 6.729E-04 | RASGRP1 |
| ENSG00000142686 | -1.80 | 6.847E-04 | C1orf216 |
| ENSG00000253792 | -3.24 | 6.861E-04 | ENSG00000253792 |
| ENSG00000212719 | 1.58 | 6.977E-04 | LINC02693 |
| ENSG00000224243 | -2.17 | 7.140E-04 | SOX1-OT |
| ENSG00000143226 | 1.88 | 7.214E-04 | FCGR2A |
| ENSG00000044459 | 1.51 | 7.247E-04 | CNTLN |
| ENSG00000185697 | -1.47 | 7.589E-04 | MYBL1 |
| ENSG00000235847 | -2.33 | 7.778E-04 | LDHAP7 |
| ENSG00000168298 | 3.33 | 7.863E-04 | H1-4 |
| ENSG00000168026 | -1.46 | 8.157E-04 | TTC21A |
| ENSG00000130511 | -1.46 | 8.190E-04 | SSBP4 |
| ENSG00000002822 | -1.42 | 8.503E-04 | MAD1L1 |
| ENSG00000278133 | -1.51 | 8.626E-04 | ENSG00000278133 |
| ENSG00000116990 | 1.45 | 8.631E-04 | MYCL |
| ENSG00000113119 | -1.53 | 8.674E-04 | TMCO6 |
| ENSG00000133958 | 1.82 | 8.843E-04 | UNC79 |
| ENSG00000139445 | -1.69 | 8.853E-04 | FOXN4 |
| ENSG00000239672 | -1.55 | 8.962E-04 | NME1 |
| ENSG00000211653 | -1.95 | 9.461E-04 | IGLV1-40 |
| ENSG00000109654 | 2.16 | 9.611E-04 | TRIM2 |
| ENSG00000175395 | 1.86 | 1.019E-03 | ZNF25 |
| ENSG00000057704 | 1.84 | 1.026E-03 | TMCC3 |
| ENSG00000239219 | -2.13 | 1.049E-03 | ENSG00000239219 |
| ENSG00000186350 | 1.45 | 1.059E-03 | RXRA |
| ENSG00000210107 | -1.54 | 1.063E-03 | MT-TQ |
| ENSG00000231503 | -1.41 | 1.068E-03 | PTMAP4 |
| ENSG00000139629 | 1.44 | 1.071E-03 | GALNT6 |
| ENSG00000171320 | -1.49 | 1.149E-03 | ESCO2 |
| ENSG00000111452 | 1.93 | 1.181E-03 | ADGRD1 |
| ENSG00000163359 | 1.65 | 1.183E-03 | COL6A3 |
| ENSG00000041982 | 2.00 | 1.189E-03 | TNC |
| ENSG00000186472 | 1.51 | 1.190E-03 | PCLO |
| ENSG00000181847 | 3.61 | 1.215E-03 | TIGIT |
| ENSG00000261512 | 1.77 | 1.236E-03 | ENSG00000261512 |
| ENSG00000249624 | -2.46 | 1.241E-03 | ENSG00000249624 |
| ENSG00000196220 | 1.60 | 1.266E-03 | SRGAP3 |
| ENSG00000197566 | 1.64 | 1.281E-03 | ZNF624 |
| ENSG00000104205 | 1.74 | 1.291E-03 | SGK3 |
| ENSG00000288018 | 1.66 | 1.299E-03 | ENSG00000288018 |
| ENSG00000108405 | 1.52 | 1.324E-03 | P2RX1 |
| ENSG00000184441 | -1.65 | 1.330E-03 | ENSG00000184441 |
| ENSG00000127423 | -1.52 | 1.333E-03 | AUNIP |
| ENSG00000135245 | -1.42 | 1.338E-03 | HILPDA |
| ENSG00000228043 | 2.63 | 1.339E-03 | SPOPL-DT |
| ENSG00000159674 | 2.15 | 1.345E-03 | SPON2 |
| ENSG00000134508 | -1.46 | 1.364E-03 | CABLES1 |
| ENSG00000213740 | -1.46 | 1.402E-03 | SERBP1P1 |
| ENSG00000113070 | -1.65 | 1.424E-03 | HBEGF |
| ENSG00000258572 | -1.61 | 1.437E-03 | ENSG00000258572 |
| ENSG00000203288 | 1.79 | 1.447E-03 | TDRKH-AS1 |
| ENSG00000188451 | 2.45 | 1.489E-03 | SRP72P2 |
| ENSG00000196787 | 1.78 | 1.531E-03 | H2AC11 |
| ENSG00000196968 | -2.82 | 1.539E-03 | FUT11 |
| ENSG00000164053 | -1.53 | 1.551E-03 | ATRIP |
| ENSG00000132881 | 1.54 | 1.574E-03 | CPLANE2 |
| ENSG00000182557 | 1.52 | 1.576E-03 | SPNS3 |
| ENSG00000211650 | -1.55 | 1.581E-03 | IGLV5-45 |
| ENSG00000169621 | 1.42 | 1.588E-03 | APLF |
| ENSG00000158887 | 1.48 | 1.603E-03 | MPZ |
| ENSG00000200795 | 3.15 | 1.611E-03 | RNU4-1 |
| ENSG00000248473 | -1.85 | 1.623E-03 | LINC01962 |
| ENSG00000248571 | -3.33 | 1.724E-03 | ENSG00000248571 |
| ENSG00000181045 | 1.54 | 1.756E-03 | SLC26A11 |
| ENSG00000231249 | 2.12 | 1.768E-03 | ITPR1-DT |
| ENSG00000165409 | -1.48 | 1.796E-03 | TSHR |
| ENSG00000197557 | -1.49 | 1.817E-03 | TTC30A |
| ENSG00000255198 | -1.93 | 1.848E-03 | SNHG9 |
| ENSG00000160183 | 1.72 | 1.865E-03 | TMPRSS3 |
| ENSG00000141391 | 1.47 | 1.969E-03 | PRELID3A |
| ENSG00000186710 | -2.46 | 1.970E-03 | CFAP73 |
| ENSG00000153404 | -1.80 | 2.037E-03 | PLEKHG4B |
| ENSG00000253540 | -1.50 | 2.071E-03 | FAM86HP |
| ENSG00000196814 | 1.61 | 2.095E-03 | MVB12B |
| ENSG00000169239 | 1.55 | 2.153E-03 | CA5B |
| ENSG00000185652 | -2.00 | 2.214E-03 | NTF3 |
| ENSG00000116678 | 1.63 | 2.223E-03 | LEPR |
| ENSG00000259959 | 1.48 | 2.246E-03 | ENSG00000259959 |
| ENSG00000267568 | -1.42 | 2.249E-03 | ENSG00000267568 |
| ENSG00000102032 | 1.46 | 2.288E-03 | RENBP |
| ENSG00000130844 | -1.42 | 2.322E-03 | ZNF331 |
| ENSG00000178038 | 1.97 | 2.325E-03 | ALS2CL |
| ENSG00000247095 | -1.96 | 2.334E-03 | MIR210HG |
| ENSG00000248243 | 2.46 | 2.379E-03 | LINC02014 |
| ENSG00000261324 | 1.59 | 2.410E-03 | ENSG00000261324 |
| ENSG00000180747 | 1.85 | 2.458E-03 | SMG1P3 |
| ENSG00000066923 | 1.43 | 2.465E-03 | STAG3 |
| ENSG00000115339 | 2.47 | 2.465E-03 | GALNT3 |
| ENSG00000286362 | 2.77 | 2.496E-03 | ENSG00000286362 |
| ENSG00000175265 | 1.76 | 2.498E-03 | GOLGA8A |
| ENSG00000198440 | 1.49 | 2.510E-03 | ZNF583 |
| ENSG00000007129 | 1.57 | 2.552E-03 | CEACAM21 |
| ENSG00000122786 | 2.65 | 2.638E-03 | CALD1 |
| ENSG00000278600 | 1.54 | 2.689E-03 | ENSG00000278600 |
| ENSG00000198208 | 1.43 | 2.739E-03 | RPS6KL1 |
| ENSG00000161677 | -2.99 | 2.742E-03 | JOSD2 |
| ENSG00000227124 | 1.44 | 2.792E-03 | ZNF717 |
| ENSG00000234719 | 1.68 | 2.797E-03 | NPIPB2 |
| ENSG00000260442 | -1.55 | 2.811E-03 | ATP2A1-AS1 |
| ENSG00000110900 | 1.43 | 2.848E-03 | TSPAN11 |
| ENSG00000287796 | 1.97 | 2.861E-03 | ENSG00000287796 |
| ENSG00000182685 | -2.31 | 2.873E-03 | BRICD5 |
| ENSG00000232053 | 2.16 | 2.874E-03 | ENSG00000232053 |
| ENSG00000186603 | -1.45 | 2.901E-03 | HPDL |
| ENSG00000062524 | 1.50 | 2.904E-03 | LTK |
| ENSG00000229043 | 1.59 | 2.921E-03 | ZFAND2A-DT |
| ENSG00000229604 | -1.54 | 2.958E-03 | MTATP8P2 |
| ENSG00000138378 | 2.34 | 2.996E-03 | STAT4 |
| ENSG00000235568 | 1.97 | 3.014E-03 | NFAM1 |
| ENSG00000235720 | -1.63 | 3.031E-03 | GABPAP |
| ENSG00000167549 | -1.73 | 3.159E-03 | CORO6 |
| ENSG00000117586 | 1.55 | 3.173E-03 | TNFSF4 |
| ENSG00000228495 | -1.51 | 3.315E-03 | LINC01013 |
| ENSG00000153208 | 1.83 | 3.320E-03 | MERTK |
| ENSG00000027001 | -1.44 | 3.341E-03 | MIPEP |
| ENSG00000261087 | 1.69 | 3.343E-03 | ZNNT1 |
| ENSG00000275793 | 2.05 | 3.395E-03 | RIMBP3 |
| ENSG00000185022 | 2.09 | 3.417E-03 | MAFF |
| ENSG00000176170 | -1.99 | 3.435E-03 | SPHK1 |
| ENSG00000170231 | -1.42 | 3.457E-03 | FABP6 |
| ENSG00000125869 | -1.63 | 3.518E-03 | LAMP5 |
| ENSG00000196975 | 1.63 | 3.617E-03 | ANXA4 |
| ENSG00000198626 | 1.51 | 3.659E-03 | RYR2 |
| ENSG00000207561 | 2.96 | 3.678E-03 | MIR635 |
| ENSG00000149260 | 1.45 | 3.681E-03 | CAPN5 |
| ENSG00000235652 | 1.53 | 3.686E-03 | EPM2A-DT |
| ENSG00000274460 | 2.54 | 3.920E-03 | ENSG00000274460 |
| ENSG00000112319 | 2.50 | 3.937E-03 | EYA4 |
| ENSG00000078081 | 1.59 | 3.950E-03 | LAMP3 |
| ENSG00000236264 | -2.73 | 3.957E-03 | RPL26P30 |
| ENSG00000224043 | -1.69 | 4.090E-03 | CCNT2-AS1 |
| ENSG00000151150 | 2.38 | 4.115E-03 | ANK3 |
| ENSG00000134042 | -1.77 | 4.156E-03 | MRO |
| ENSG00000135898 | 2.10 | 4.262E-03 | GPR55 |
| ENSG00000285534 | 1.50 | 4.394E-03 | ENSG00000285534 |
| ENSG00000198478 | 1.78 | 4.402E-03 | SH3BGRL2 |
| ENSG00000188818 | -1.43 | 4.507E-03 | ZDHHC11 |
| ENSG00000288596 | 1.66 | 4.554E-03 | C8orf44 |
| ENSG00000285802 | -2.52 | 4.683E-03 | ENSG00000285802 |
| ENSG00000227704 | -1.70 | 4.738E-03 | ENSG00000227704 |
| ENSG00000137274 | -1.50 | 4.823E-03 | BPHL |
| ENSG00000189337 | 1.47 | 4.860E-03 | KAZN |
| ENSG00000273221 | 1.93 | 4.874E-03 | ENSG00000273221 |
| ENSG00000211651 | -2.16 | 4.874E-03 | IGLV1-44 |
| ENSG00000167077 | 1.45 | 4.878E-03 | MEI1 |
| ENSG00000271503 | 1.62 | 4.976E-03 | CCL5 |
| ENSG00000255337 | 2.01 | 5.164E-03 | TMEM123-DT |
| ENSG00000283403 | -1.56 | 5.222E-03 | ENSG00000283403 |
| ENSG00000107738 | 1.77 | 5.244E-03 | VSIR |
| ENSG00000154874 | 1.47 | 5.367E-03 | CCDC144B |
| ENSG00000183647 | -1.64 | 5.369E-03 | ZNF530 |
| ENSG00000157782 | 2.18 | 5.449E-03 | CABP1 |
| ENSG00000102409 | 1.49 | 5.645E-03 | BEX4 |
| ENSG00000243742 | 1.93 | 5.649E-03 | RPLP0P2 |
| ENSG00000219626 | 1.48 | 5.671E-03 | FAM228B |
| ENSG00000188452 | 2.65 | 5.746E-03 | CERKL |
| ENSG00000231991 | -1.46 | 5.784E-03 | ANXA2P2 |
| ENSG00000223881 | 1.71 | 5.871E-03 | ENSG00000223881 |
| ENSG00000140465 | 2.45 | 6.018E-03 | CYP1A1 |
| ENSG00000001617 | -1.57 | 6.044E-03 | SEMA3F |
| ENSG00000213231 | -1.89 | 6.103E-03 | TCL1B |
| ENSG00000161714 | -1.44 | 6.509E-03 | PLCD3 |
| ENSG00000231721 | 1.44 | 6.523E-03 | LINC-PINT |
| ENSG00000272989 | -2.27 | 6.561E-03 | LINC02012 |
| ENSG00000122863 | 2.83 | 6.562E-03 | CHST3 |
| ENSG00000154957 | 1.58 | 6.616E-03 | ZNF18 |
| ENSG00000185250 | 1.73 | 6.627E-03 | PPIL6 |
| ENSG00000255121 | -1.42 | 6.689E-03 | CENATAC-DT |
| ENSG00000241478 | -2.48 | 6.698E-03 | HSPA8P9 |
| ENSG00000174945 | 1.99 | 6.781E-03 | AMZ1 |
| ENSG00000211659 | -1.90 | 6.952E-03 | IGLV3-25 |
| ENSG00000287065 | 2.49 | 6.988E-03 | ENSG00000287065 |
| ENSG00000162669 | -2.20 | 7.320E-03 | HFM1 |
| ENSG00000173110 | 2.42 | 7.485E-03 | HSPA6 |
| ENSG00000225335 | 2.61 | 7.507E-03 | ENSG00000225335 |
| ENSG00000287625 | 2.54 | 7.577E-03 | ENSG00000287625 |
| ENSG00000242759 | 1.46 | 7.582E-03 | LINC00882 |
| ENSG00000064199 | -1.50 | 7.691E-03 | SPA17 |
| ENSG00000289635 | -1.54 | 7.716E-03 | ENSG00000289635 |
| ENSG00000244039 | -2.09 | 7.768E-03 | ENSG00000244039 |
| ENSG00000280079 | -1.76 | 7.854E-03 | ENSG00000280079 |
| ENSG00000004660 | 1.58 | 7.985E-03 | CAMKK1 |
| ENSG00000232859 | 2.04 | 8.027E-03 | LYRM9 |
| ENSG00000261123 | 1.74 | 8.149E-03 | ENSG00000261123 |
| ENSG00000289020 | -1.60 | 8.164E-03 | ENSG00000289020 |
| ENSG00000245293 | 1.62 | 8.193E-03 | CYP2U1-AS1 |
| ENSG00000166126 | -1.41 | 8.300E-03 | AMN |
| ENSG00000131042 | -2.41 | 8.373E-03 | LILRB2 |
| ENSG00000243660 | 1.57 | 8.431E-03 | ZNF487 |
| ENSG00000176490 | 2.36 | 8.542E-03 | DIRAS1 |
| ENSG00000211666 | -1.56 | 8.653E-03 | IGLV2-14 |
| ENSG00000267383 | -1.45 | 8.687E-03 | ENSG00000267383 |
| ENSG00000176658 | 1.45 | 8.784E-03 | MYO1D |
| ENSG00000179630 | 1.53 | 8.835E-03 | LACC1 |
| ENSG00000169129 | 1.70 | 8.866E-03 | AFAP1L2 |
| ENSG00000162711 | 2.28 | 8.894E-03 | NLRP3 |
| ENSG00000123685 | 1.67 | 9.002E-03 | BATF3 |
| ENSG00000178814 | 2.64 | 9.072E-03 | OPLAH |
| ENSG00000163378 | 1.65 | 9.194E-03 | EOGT |
| ENSG00000289396 | 2.94 | 9.215E-03 | ENSG00000289396 |
| ENSG00000163590 | 1.80 | 9.280E-03 | PPM1L |
| ENSG00000230303 | -2.30 | 9.423E-03 | ENSG00000230303 |
| ENSG00000203724 | 2.70 | 9.436E-03 | C1orf53 |
| ENSG00000254416 | 2.50 | 9.512E-03 | LINC02732 |
| ENSG00000229124 | 2.07 | 9.774E-03 | VIM-AS1 |
| ENSG00000269821 | 1.49 | 9.857E-03 | KCNQ1OT1 |
| ENSG00000233030 | 2.86 | 9.888E-03 | ENSG00000233030 |
| ENSG00000237232 | -2.57 | 9.922E-03 | ZNF295-AS1 |
| ENSG00000238287 | 2.00 | 9.998E-03 | EXO5-DT |

**Supplementary Figure 1. Canonical Pathways identified by Ingenuity Pathway Analysis sorted by -log10 p-values**. The threshold indicates a p-value of 0.01 (Fisher’s Exact Test). The ratio is the number of differentially expressed genes in the Namalwa cells treated with PFOA over the total number of genes involved in each pathway. The total number of genes involved in each pathway is indicated above the bar charts.

**Supplementary Figure 2. Effect of exposure to AS1842856 on viability of Namalwa cells.** Namalwa cells were exposed to 0.01 or 0.1 µM AS1842856 for 48 h. Viability was determined using the WST-1 assay and expressed as percentage of solvent control (0.1% DMSO). Data presented as mean ± SD from 18 wells for solvent control and for 6 wells for AS1842856-exposed cells (data from two independent runs).
